# Contribution of vaccination to improved survival and health: modelling 50 years of the Expanded Programme on Immunization

**DOI:** 10.1016/S0140-6736(24)00850-X

**Published:** 2024-05-25

**Authors:** Andrew J Shattock, Helen C Johnson, So Yoon Sim, Austin Carter, Philipp Lambach, Raymond C W Hutubessy, Kimberly M Thompson, Kamran Badizadegan, Brian Lambert, Matthew J Ferrari, Mark Jit, Han Fu, Sheetal P Silal, Rachel A Hounsell, Richard G White, Jonathan F Mosser, Katy A M Gaythorpe, Caroline L Trotter, Ann Lindstrand, Katherine L O'Brien, Naor Bar-Zeev

**Affiliations:** a Swiss Tropical and Public Health Institute, Basel, Switzerland; b University of Basel, Basel, Switzerland; c Telethon Kids Institute, Perth, Australia; d University of Western Australia, Perth, Australia; e Safinea, London, UK; f London School of Economics and Political Science, London, UK; g London School of Hygiene & Tropical Medicine, London, UK; h University of Cambridge, Cambridge, UK; i World Health Organization, Geneva, Switzerland; j University of Washington, Seattle, WA, USA; k Kid Risk, Orlando, FL, USA; l Pennsylvania State University, University Park, PA, USA; m Modelling and Simulation Hub, Africa, Department of Statistical Sciences, University of Cape Town, Cape Town, South Africa; n Centre for Global Health, Nuffield Department of Medicine, Oxford University, Oxford, UK; o Imperial College London, London, UK; p TB Modelling Group, Infectious Disease Epidemiology Department, London School of Hygiene & Tropical Medicine, London, UK

## Abstract

**Background:**

WHO, as requested by its member states, launched the Expanded Programme on Immunization (EPI) in 1974 to make life-saving vaccines available to all globally. To mark the 50-year anniversary of EPI, we sought to quantify the public health impact of vaccination globally since the programme's inception.

**Methods:**

In this modelling study, we used a suite of mathematical and statistical models to estimate the global and regional public health impact of 50 years of vaccination against 14 pathogens in EPI. For the modelled pathogens, we considered coverage of all routine and supplementary vaccines delivered since 1974 and estimated the mortality and morbidity averted for each age cohort relative to a hypothetical scenario of no historical vaccination. We then used these modelled outcomes to estimate the contribution of vaccination to globally declining infant and child mortality rates over this period.

**Findings:**

Since 1974, vaccination has averted 154 million deaths, including 146 million among children younger than 5 years of whom 101 million were infants younger than 1 year. For every death averted, 66 years of full health were gained on average, translating to 10·2 billion years of full health gained. We estimate that vaccination has accounted for 40% of the observed decline in global infant mortality, 52% in the African region. In 2024, a child younger than 10 years is 40% more likely to survive to their next birthday relative to a hypothetical scenario of no historical vaccination. Increased survival probability is observed even well into late adulthood.

**Interpretation:**

Since 1974 substantial gains in childhood survival have occurred in every global region. We estimate that EPI has provided the single greatest contribution to improved infant survival over the past 50 years. In the context of strengthening primary health care, our results show that equitable universal access to immunisation remains crucial to sustain health gains and continue to save future lives from preventable infectious mortality.

**Funding:**

WHO.

## Introduction

The Expanded Programme on Immunization (EPI) was established by the World Health Assembly in May, 1974, marking a proactive commitment to extend the protective benefits of vaccination to all.^1^ Motivated by successful progress towards smallpox eradication, a milestone achieved in 1980, WHO launched the collaborative initiative with the initial goal to vaccinate all children against smallpox, tuberculosis, diphtheria, tetanus, pertussis, poliomyelitis, and measles by 1990.^2^ EPI now also includes protection against other global and regional specific pathogens, across all ages of the life course, whose inclusion is determined by country programme decisions (panel). Since 1974, this growth in the number of diseases covered by vaccination programmes, coupled with catalytic strategies and initiatives, and underpinned by a vision shared by the global community, achieved massive scale-up in breadth of protection and coverage. Global coverage with a third dose of the diphtheria–tetanus–pertussis (DTP3) vaccine, a proxy for vaccine programme performance, increased from less than 5% in 1974 to 86% in 2019 before the COVID-19 pandemic, and is now 84%.^15^

Panel **Timeline of key milestones that increased global access to vaccines**

**1974: WHO Expanded Programme on Immunization (EPI)**

The 27th World Health Assembly resolution formally established EPI against diphtheria, pertussis, tetanus, measles, poliomyelitis, tuberculosis, smallpox, and other diseases, where applicable, according to country-specific epidemiological situation.^1^

**1979: Pan American Health Organization (PAHO) revolving fund**

The Pan American Sanitary Conference resolution established the working capital for the PAHO revolving fund, a mechanism facilitating pooled procurement and increasing access to vaccines, syringes, and cold-chain equipment at affordable prices.^3^

**1982: UNICEF Child Survival and Development Revolution**

UNICEF launched the Child Survival and Development Revolution with a focus on four measures: growth monitoring, oral rehydration therapy, promotion of breastfeeding, and immunisation (known as GOBI).^4^

**1984: EPI's first standardised schedule**

EPI revised WHO's 1961 standardised schedule, and included vaccinations for tuberculosis (BCG vaccine at birth), diphtheria, tetanus, and pertussis (DTP) and poliomyelitis vaccinations (DTP and poliomyelitis at 6, 10, and 14 weeks), and measles vaccination (at 9 months).^5^

**1990: Declaration of Manhattan, Children's Vaccine Initiative**

The Children's Vaccine Initiative aimed to accelerate efforts to develop vaccines that could enhance the performance of EPI.^6^

**1999: The Strategic Advisory Group of Experts (SAGE) on immunisation**

SAGE was established by the Director-General of WHO to advise WHO on overall global policies and strategies, ranging from vaccines and technology, research and development, to delivery of immunisation and its links with other health interventions.^7^

**2000: Gavi, The Vaccine Alliance**

Gavi, the Vaccine Alliance (previously GAVI) was established as a public–private partnership to address market failure in selected countries and accelerate equal access to new and under-utilised vaccines.^8^

**2000 to present: ongoing acceleration of new vaccine introduction**

• Accelerated Development and Introduction Plans for pneumococcal conjugate vaccines (PCV) and rotavirus vaccines and the *Haemophilus influenzae* type B Initiative expedited vaccine introduction in Gavi-supported countries.^9^
• The pneumococcal Advance Market Commitment contributed to scaling up PCV supply and coverage.^10^
• The Meningitis Vaccine Project led to development, testing, licensure, and introduction of a meningococcal A conjugate vaccine (ie, MenAfriVac).^11^
• The Malaria Vaccine Implementation Programme evaluated the public health use of the RTS,S malaria vaccine and informed the first WHO SAGE recommendation for a malaria vaccine.^12^

**2017: Coalition for Epidemic Preparedness Innovations (CEPI)**

As a global response to Ebola virus, Zika virus, and severe acute respiratory syndrome (known as SARS) outbreaks, CEPI was launched to develop safe and effective vaccines for emerging infectious diseases to prevent future epidemics.

**2020: Immunization Agenda 2030 (IA2030)**

Building on lessons learned from the Global Immunization Vision and Strategy (2006–15) and the Global Vaccine Action Plan (2011–20), IA2030 was endorsed by the 73rd World Health Assembly in August, 2020; IA2030 advances the commitment set by EPI and global initiatives to ensure universal access to vaccines, strengthening primary health care and supporting universal health coverage.

**2020–23: COVID-19 Vaccines Global Access (COVAX)**

COVAX was the vaccine pillar of the Access to COVID-19 Tools (known as ACT) Accelerator partnership, established to speed up development, production, and equitable distribution of COVID-19 tests, treatments, and vaccines, reducing COVID-19 mortality and severe diseases and restoring full societal and economic activity.^13^

**2023–24: The Big Catch-Up**

The Big Catch-Up initiative aims to restore immunisation coverage to pre-COVID-19 pandemic levels, catch-up children whose doses were missed because of the pandemic, and strengthen routine immunisation systems to achieve 2030 targets.^14^

**2024: EPI expansion**

EPI expanded to cover vaccines against 13 vaccine-preventable diseases across the life course at the global level (tuberculosis, COVID-19, diphtheria, hepatitis B, *H influenzae* type B, human papillomavirus, measles, rubella, invasive pneumococcal disease, pertussis, poliomyelitis, rotavirus, and tetanus) and over 17 context-specific vaccine-preventable diseases (including cholera, dengue, hepatitis A, influenza, Japanese encephalitis, malaria, meningitis, mpox, mumps, rabies, respiratory syncytial virus, typhoid, tick-borne encephalitis, varicella, yellow fever, and zoster).^5^

**Ongoing disease eradication and elimination initiatives**

Since WHO's declaration of smallpox eradication in 1980, nine eradication and elimination strategies have been established: The Global Polio Eradication Initiative (1988), Maternal and Neonatal Tetanus Elimination (1989), The Measles & Rubella Initiative (2001), The End TB strategy (2015), The Global Health Sector Strategy on Viral Hepatitis (2016), The Global Technical Strategy For Malaria (2016), The Eliminate Yellow Fever Epidemics Strategy (2017), The Global Strategy to Accelerate the Elimination of Cervical Cancer (2020), and The Global Roadmap to Defeat Meningitis (2020).^5^

## Methods

### Study design

In this modelling study, to quantify the impact of EPI, we estimated the number of deaths averted, the life-years gained, and the years of full health gained (ie, disability-adjusted life-years averted) by vaccination (henceforth vaccine impact) against 14 pathogens (ie, diphtheria, *Haemophilus influenzae* type B, hepatitis B, Japanese encephalitis, measles, meningitis A, pertussis, invasive pneumococcal disease, poliomyelitis, rotavirus, rubella, tetanus, tuberculosis, and yellow fever) in 194 WHO member states between June 1, 1974, and May 31, 2024, through coverage achieved by routine and supplementary immunisation activities. We developed a standardised analytical framework to estimate vaccine impact per fully vaccinated person over time, synthesising the results of 22 models and applying regression-based imputation methods to ensure geographical and temporal completeness. We also estimated the attributable contribution of vaccination to the reduction in infant mortality from 1974 to 2024 and the regional variation in the absolute and relative impact of vaccination.

**Research in context**

**Evidence before this study**

We searched PubMed on March 13, 2024, without date limits, using the search terms “((((vaccine) AND (impact)) AND (model)) AND (countries)) AND (mortality OR morbidity)”. Studies were included if they used modelling approaches to estimate the health impact of vaccination in multiple countries for at least one pathogen that is covered by the Expanded Programme on Immunization (EPI). Of 1268 results, 87 studies met the inclusion criteria. 81 of these studies focused on the impact of a single vaccine in more than one country. Six studies considered the impact of multiple vaccines with a broader geographical scope. Of these, five studies were published by the Vaccine Impact Modelling Consortium. Two studies estimated the number of deaths and disability-adjusted life-years averted due to vaccines against ten pathogens supported by Gavi, the Vaccine Alliance in low-income and middle-income countries between 2000 and 2030. The other three studies focused on the lasting impact of COVID-19-related disruptions on routine and non-routine immunisation services, the implications of recovery and catch-up, and the effect of different recovery scenarios in these countries. One study by WHO and its partners estimated the potential impact of reaching the aspirational coverage targets of the Immunization Agenda 2030 for vaccines against 14 pathogens between 2021 and 2030 in 194 countries. No study existed that estimated the global impact of EPI since its commencement.

**Added value of this study**

This study is the most comprehensive modelling analysis of historical vaccine impact to date. The study covers 14 pathogens over a 50-year timeframe (1974–2024) at the global level (194 WHO member states). Furthermore, the analysis advances the previous work by Carter and colleagues, in 2023, by incorporating additional sources of coverage estimates for non-routine immunisation activities, improving characterisation of disease epidemiology for the static component of modelling, and capturing the impact of vaccination on reducing morbidity in addition to mortality. This study contributes to the existing literature on global vaccination impact modelling by developing novel approaches to synthesising diverse sources of model estimates, accounting for non-linearity in vaccine impact, and extrapolating model outputs to locations without such estimates.

**Implications of all the available evidence**

Vaccination since 1974 has made the greatest contribution of any health intervention to mortality reduction and years of full health gained. The results from this study show the impact of vaccination on infant and child mortality and morbidity reduction over the last 50 years, and that the protective effects persist throughout the life course. The greatest contribution is due to measles vaccination. The substantial gains identified in childhood survival due to vaccination highlight the importance of immunisation as a crucial part of primary health care. Ongoing political commitment, sustainable investment, and maintaining local capacity to strengthen health systems will protect the gains of past decades and sustain them into the future. Ensuring these benefits are extended further to reach unvaccinated and under-vaccinated children and missed communities, especially with the measles vaccine, will be crucial to maximise the number of future lives saved. Additional vaccines such as the human papillomavirus vaccine and malaria vaccines are likely to increase further the life-saving impact of immunisation programmes.

### Procedures

We provide here brief details regarding the suite of mathematical and statistical models; a more complete description is given in the appendix (pp 11–43).

We synthesised age-specific vaccine coverage estimates from four data sources: WHO Immunization dashboard (for routine activities); WHO Supplementary Immunization Activities Database; WHO Polio Information System (for supplementary immunisation activities); and Vaccine Impact Modelling Consortium (VIMC) coverage estimates.^16^, ^17^, ^18^ Where country coverage data between 1974 and 1979 were unavailable, for low-income and middle-income countries we linearly extrapolated from known coverage in 1980 to an anchored 0% coverage in 1974, for high-income countries we applied the coverage reported in 1980 to this period (appendix pp 19–20). In total, we evaluated 24 vaccine activities (stratifying each disease, vaccine, and dose number; and routine or supplementary; such that measles dose 1 provided as part of routine immunisation is a distinct activity from measles dose 1 given as part of a vaccination campaign, and both differ from measles dose 2 or from vaccinations for other pathogens), calculating the number of fully vaccinated people using population estimates from World Population Prospects.^19^

Modelling took three forms. First, impact estimates were derived directly through simulation of published transmission models for measles and poliomyelitis in all 194 WHO member states for the full 50-year analysis period. For measles an ensemble of two published dynamic models was used.^20^, ^21^ For poliomyelitis we ran novel simulations of an existing dynamic model.^22^ Second, we extended a suite of VIMC transmission models for *H influenzae* type B, hepatitis B, Japanese encephalitis, invasive pneumococcal disease, rotavirus, and rubella, which estimated vaccine impact for 110 countries (fewer for meningitis A and yellow fever) from 2000 to 2024, by geographical imputation and temporal extrapolation.^18^ Finally, published static disease burden models for diphtheria, tetanus, pertussis, and tuberculosis were upgraded (appendix pp 23–31).^23^ For these static models we incorporated estimates reported by the 2021 Global Burden of Disease (GBD) study using three key metrics: GBD-estimated country-specific and age-specific disease-attributable mortality and morbidity; vaccine efficacy (interpreted as the reduction in probability of death or disease) profiles, including effects of waning immunity, which were also specifically extrapolated for priming, boosting, pregnancy schedules, and vaccine platforms (eg, acellular and whole-cell pertussis); and country-specific and age-specific vaccine coverage.^24^ Vaccine efficacy and vaccine coverage were combined to produce an estimate of effective vaccine coverage, which was then used to estimate disease-attributable mortality and morbidity in a hypothetical scenario of no historical vaccination for the nine vaccines considered (appendix pp 25–26). All forms of modelling allowed us to capture both individual effects of vaccines (ie, protecting the vaccinated) and population-level effects (ie, reducing transmission and incidence, and indirectly protecting the unvaccinated; appendix pp 38–42). When extending existing models, we compared the results against those of previously conducted analyses that were restricted in time and space, parsing the findings accordingly.

### Outcomes

The primary outcome of this study was to quantify the impact of EPI from 1974 to 2024 on deaths averted, years of life gained, and years of full health gained, and to estimate the proportion of infant (younger than 1 year) mortality reduction attributable to vaccination. As a secondary outcome, we sought to evaluate these outcomes by WHO region and World Bank income stratum.

### Statistical analysis

To impute vaccine impact in countries outside of the scope of the VIMC, we fitted time series regression models with the outcome of deaths averted and years of full health gained for each vaccine in each country where VIMC estimates were available. Time series regression models regress each timepoint of an outcome variable (for this study, timepoints were defined as years) against the same or lagged timepoints of predictor variables. We used a corrected Akaike Information Criterion model selection approach to inform the choice of socioeconomic and demographic covariates, selecting the parsimonious model with best performance, on average, for each region and pathogen. Using the regional median coefficient for each included predictor variable, combined with local data, we used the selected model to impute the impact in countries with missing data (appendix pp 33–38). To estimate vaccine impact in time periods not directly modelled, we fitted a functional relationship between model-estimated cumulative impact—in terms of either deaths averted or years of full health gained—and the cumulative number of fully vaccinated people. Four functional forms were fitted for each vaccine in each country: linear (presumes each dose has equal effect, no community herd effect), logarithmic, exponential (each additional dose has a respectively lesser or greater effect), and sigmoidal (programme takes time to establish and achieve community effects, then each subsequent dose has less individual effect). Therefore we selected functions that best fit locally specific data, thereby capturing locally relevant interactions between the individual and population effects of specific vaccines at specific places and times.

A Markov chain Monte Carlo algorithm was used to derive posteriors for all fitted function parameters. The Akaike Information Criterion was then used to select the most appropriate functional form for each vaccine in each country. Using these fitted relationships between vaccine coverage (in terms of fully vaccinated people) and vaccine impact (in terms of deaths averted or years of full health gained), vaccine impact was inferred either backward or forward in time according to observed coverage in all cases where modelled estimates were not available. The parameter posteriors generated through this functional fitting process were used to propagate uncertainty for final vaccine impact estimates (appendix pp 2–6, 9–10). Vaccine coverage and population size sources used are not statistical estimators per se and uncertainty bounds for these are not reported; we took them as provided. Propagation of uncertainty at all levels of estimation was also not possible for all the hierarchical underlying models or for the values input into those models. For the modelling in this study, posterior credible intervals were produced, but these were of arbitrarily predefined widths and should not be interpreted as bounds for the final reported outcomes (appendix pp 42–43).

For all studied pathogens, we estimated the deaths averted by age (0–100 years) for each studied year (1974–2024). For measles, poliomyelitis, and the VIMC models years of full health gained were estimated using the modelled number of cases of specific severity, along with disease burden disability weights provided by GBD 2021.^24^ For the static models of diphtheria, pertussis, tetanus, and tuberculosis, an approach identical to that for computing deaths averted was used, but with corresponding GBD estimates for disease burden. Age granularity was derived using linear interpolation from 5-year bins as provided by GBD. These age-specific results were then directly used along with life expectancy values to estimate years of life saved. We used country-stratified and year-stratified life expectancy values from World Population Prospects for this study.^19^ The risk of double counting (one individual's death being averted for multiple diseases) was anticipated and addressed using a Bernoulli approach (appendix p 43).

Using population and all-cause mortality estimates from World Population Prospects along with our modelled estimates of deaths averted, we estimated infant (younger than 1 year) mortality rates in the hypothetical scenario of no vaccination since 1974. Furthermore, by considering a second hypothetical scenario of no decline in infant mortality rates over the analysis period, we estimated the extent to which the observed global and regional decline in infant mortality since 1974 was attributable to vaccination. We assumed that the infant population would be of the same size in all scenarios; meaning that, in scenarios of higher infant mortality, birth rates were modelled to be higher, so that the infant population was stable across all models.

Finally, we assessed age-disaggregated absolute and relative survival gains achieved through vaccination both globally and regionally for individuals alive in 2024. That is, the increase in the probability of 1-year survival due to global vaccination activities since 1974 for an individual of a given age. This metric was estimated by considering the difference between age-specific mortality rates in 2024 in the real-life scenario compared with the hypothetical no historical vaccination scenario.

These methods were reviewed by WHO's Immunization and Vaccines Related Implementation Research Advisory Committee.^25^

### Role of the funding source

The funder of the study had no role in study design, data collection, data analysis, data interpretation, or writing of the report.

## Results

**Figure 1:**
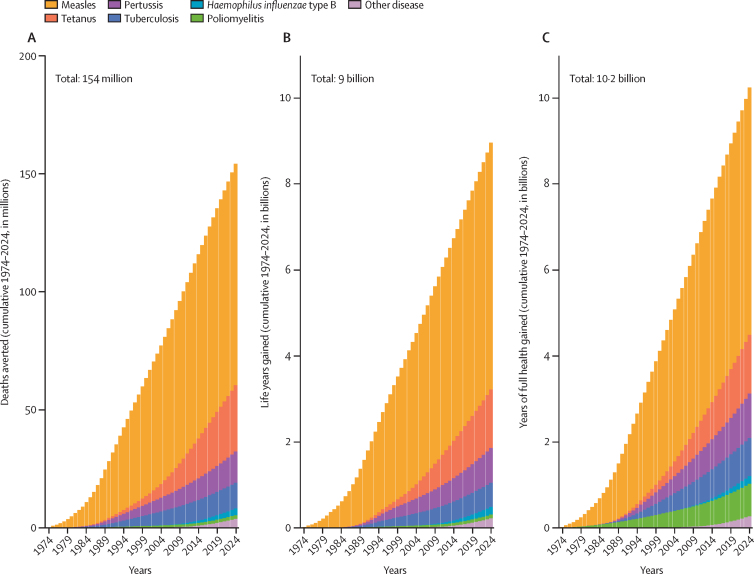
Deaths averted, years of life saved, and years of full health gained due to vaccination Data are cumulative 1974–2024. Measles: deaths averted: 93·7 million; years of life saved: 5·7 billion; years of full health gained: 5·8 billion. Tetanus: deaths averted: 27·9 million; years of life saved: 1·4 billion; years of full health gained: 1·4 billion. Pertussis: deaths averted: 13·2 million; years of life saved: 0·8 billion; years of full health gained: 1 billion. Tuberculosis: deaths averted: 10·9 million; years of life saved: 0·6 billion; years of full health gained: 0·9 billion. Haemophilus influenzae type B: deaths averted: 2·8 million; years of life saved: 0·2 billion; years of full health gained: 0·2 billion. Poliomyelitis: deaths averted: 1·6 million; years of life saved: 0·1 billion; years of full health gained: 0·8 billion. Other diseases: deaths averted: 3·8 million; years of life saved: 0·2 billion; years of full health gained: 0·3 billion.

Between June 1, 1974, and May 31, 2024, vaccination programmes targeting the 14 modelled pathogens were estimated to have averted 154 million deaths (figure 1A), including 146 million in children younger than 5 years, among whom 101 million were infants younger than 1 year. 9·0 billion life-years were gained (figure 1B), along with 10·2 billion years of full health (ie, disability-adjusted life-years averted; figure 1C): over 200 million healthy life-years gained per year globally. For each life saved, an average of 58 years of life and 66 years of full health were gained, with 0·8 billion (7·8%) of the 10·2 billion years of full health gained attributable to poliomyelitis cases averted. Overall, measles vaccination accounted for 93·7 million lives saved (60·8%) of the 154·0 million total lives saved over this 50-year period. Measles vaccination was the single greatest driver of lives saved by vaccination, across all years in every region and all World Bank income strata (appendix pp 2–7).

**Figure 2:**
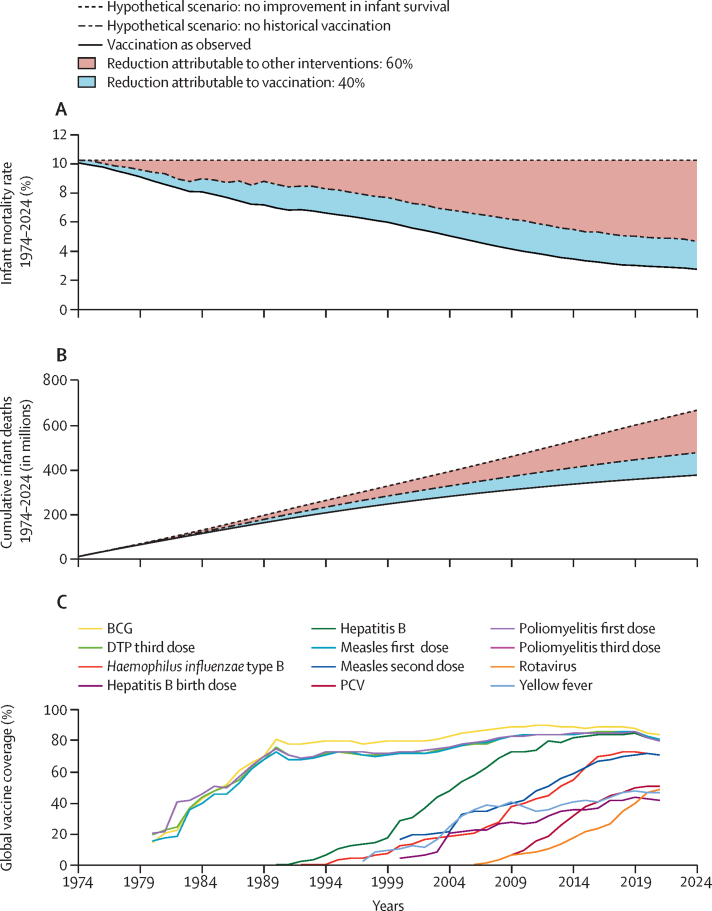
Infant mortality 1974–2024, the proportional effect of vaccination on overall decreasing trends, and global vaccine coverage DTP=diphtheria–tetanus–pertussis. PCV=pneumococcal conjugate vaccine.

Since 1974, global infant mortality has declined substantially (figure 2A). Vaccination was estimated to be directly responsible for 40% of this achievement, varying from 21% in the Western Pacific region to 52% in the African region (figure 2A, B; appendix p 8). The relative contribution to global infant mortality was especially high during the 1980's, a period of intense scale-up of coverage of the original EPI vaccines: BCG, DTP, measles, and poliomyelitis vaccines (figure 2C).

**Figure 3:**
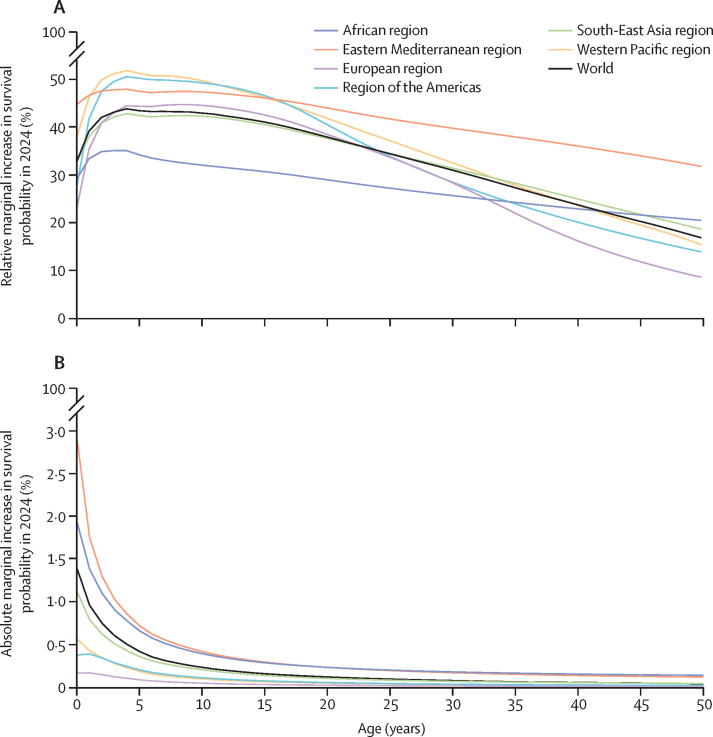
Marginal increase in survival probability in 2024 by year of life and WHO Region, compared with the hypothetical scenario of no historical vaccination Relative represents proportional percent change in this baseline risk. Absolute represents percentage point reduction in 2024 risk of death for the next year for a person of a given age. (A) Relative marginal increase in survival probability in 2024. (B) Absolute marginal increase in survival probability in 2024.

We estimate that, in 2024, children aged 10 years are approximately 44% more likely to survive to their next birthday than if no vaccinations had occurred since 1974, individuals aged 25 years are 35% more likely, and those aged 50 years are 16% more likely (figure 3A). In terms of absolute impact, the Eastern Mediterranean and African regions have seen the largest vaccine-induced gains in life course survival probability since 1974, with the European region seeing the lowest absolute gains (figure 3B). Conversely, in terms of relative impact, the Western Pacific and European regions have seen the largest gains in life course survival probability, and the African region is among the lowest, given a higher burden of competing risks (figure 3A).

As part of method development, we found that many of our models fit the local data better when the function presumed community effects (appendix p 39), although this varied by pathogen (appendix pp 9–10, 39). When comparing our models to previous, more spatiotemporally restricted uses, the results of this study fell within existing, published error margins. We calculated that the risk of double counting would have a small (0·01%) effect on our overall estimates (appendix p 43).

## Discussion

On the 50th anniversary of EPI, we present the most comprehensive assessment of historical vaccine programme impact. The vaccines modelled in this study are estimated to have saved 154 million lives since 1974, 95% of these in children younger than 5 years. This equates to 9·0 billion life-years saved and, further considering the added benefit of reduced morbidity, 10·2 billion healthy years of life have been gained due to vaccination. Measles vaccination has been the single greatest contributor and is likely to remain so.^18^, ^23^ Vaccination has accounted for close to half the total global reduction in infant mortality, and in some regions to the majority of these gains (appendix p 8). As a result of 50 years of vaccination, a child born today has a 40% increase in survival for each year of infancy and childhood. The survival benefits of infant vaccination extend to beyond 50 years of age, a remarkable finding considering the exclusion of smallpox and the exclusion of the anticipated benefits of human papillomavirus (HPV), influenza, SARS-CoV-2, Ebola, mpox and other vaccines affecting adult mortality.

Many vaccines protect in two ways, by direct reduction of risk to the vaccinated individual and, for most vaccines (although notably not tetanus), by reducing the community transmission and by reducing exposure to infectious diseases. Paradoxically, as vaccination programmes reduce community transmission, the measurable marginal direct individual benefit of vaccination becomes more modest since there is less circulating disease to prevent. We accounted for both the individual and communal benefits of vaccine programmes and their complex non-linear interactions. The observation that many models fitted local data better when the model function incorporates community effects suggests that even small reductions in community vaccine coverage can result in substantial increased risk of disease, which further work will explore. Indeed, a worldwide resurgence of large measles outbreaks is under way, consequent to pandemic-associated declines in measles vaccine coverage.^26^, ^27^ Measles outbreaks are a tracer for vaccine programme performance under the Immunization Agenda 2030 (panel). Historically, the impact of measles vaccination on annual mortality reduction peaked contemporaneously with global scale up of first dose coverage. Vaccine coverage then plateaued (figure 2C), while other non-vaccine factors that reduce infant and child mortality were introduced (figure 2A), although this varies by region (panel; appendix pp 9–10). These non-vaccine factors also contributed to lowering the risk of dying from measles, given infection. Despite the importance of non-vaccine factors, forecasting suggests that measles vaccination will remain the pre-eminent intervention that will maximise lives saved well into the future.^23^, ^27^ In the 21st century, the increasing effect of other interventions is notable, highlighting the need for sustained investment and implementation efforts, bringing together immunisation and primary health-care services.

Unlike measles vaccination, which breaks communal chains of transmission, tetanus vaccinations only protect the vaccinated individual or newborns through placental transfer of immunity. Absence of plateauing population-level effects means per-dose impact remains high (appendix pp 9–10). Tetanus elimination in mothers and newborns can be achieved through concerted effort to achieve sufficient, timely access to immunisation for pregnant women and their newborns, leading to large relative reductions in newborn disease. Pertussis vaccination was a major contributor to lives saved. Nevertheless, pertussis mortality remains a persistent preventable cause of death in young infants in all settings. In many settings the acellular vaccine is used since it is less reactogenic than the whole-cell vaccine, but it is now known to provide less durable protection, making booster doses in pregnancy important. The contribution of tetanus and pertussis highlights the importance of immunisation programmes for pregnant women. Strengthening and extending these programmes to include influenza, respiratory syncytial virus and group B Streptococcus provide further opportunities for saving future lives, and efficacious and sufficiently powered safety studies of prenatal delivery can support increased adherence. Despite being the oldest and most ubiquitously used vaccine, neonatal BCG vaccine impact on tuberculosis mortality was modest. This finding is explained by the vaccine's low biological efficacy, which varies by strain, and the probable waning efficacy by adulthood.^28^ New-generation tuberculosis vaccines are in development.^29^ This analysis did not include the putative effects of the BCG or measles vaccine on mortality from causes other than tuberculosis and measles, which some evidence suggests could be substantial. Vaccination against poliomyelitis has had a modest impact on mortality, averting 1% of deaths, but has led to substantial public health gains by reducing poliomyelitis-induced paralysis, accounting for 8% of the 10·8 billion healthy life-years gained. The opportunity to eradicate this long-standing disease, as was done with smallpox, must not be missed. The closer we get to poliomyelitis eradication the greater the challenge to reach it, but so is the commensurate obligation to complete the task.

The study found that, in 2024, both children and adults are more likely to survive to their next birthday than if no vaccinations had occurred since 1974. These results highlight the continued positive effect of vaccination throughout the life course, even in the context of waning vaccine immunity and an analysis focused on infant-specific and child-specific schedules, which does not include other vaccination programmes; such as for HPV, influenza, or COVID-19; which specifically reduce adult mortality.

A secondary aim of this study was to evaluate vaccine impact by region and other predictors. We found that larger absolute gains occurred in regions with initially high mortality, although relative benefit has been lower in such areas, because of competing mortality risks. Vaccines promote equity by saving more lives in places where more deaths occur. The contribution of vaccines to the total reduction in infant mortality varied across regions, being higher in the WHO African and European regions, regions in which the absolute mortality burdens are quite different. Correctly interpreting such findings requires consideration of both the relative and absolute effects. In both Africa and Europe vaccines have contributed to a substantial proportion of the reduced infant mortality, but in Africa this has meant many more lives are saved in absolute terms, showcasing the high vaccine impact attainable in regions with the highest infectious disease burden. Over the life course, EPI vaccines increase current survival probability at every age in Africa in both relative and absolute terms, but in people born more recently the measurable impact is less than among those born in earlier decades. This is consistent with the finding that, despite the enormous contribution of vaccines for infant survival, in recent years non-vaccine interventions are saving an increasing proportion of lives. The Immunisation Agenda 2030^26^ places vaccination squarely within the remit of primary health care and the Alma Ata Declaration. Vaccine programmes are often the backbone for systems that provide other life-saving health-care delivery. The present authors plan to extend our analyses to examine the effect of sociodemographic factors on the achievable impact of vaccination programmes and examine underlying explanatory differences across and within regions. The analysis presented here is a minimum conservative estimate of vaccine impact. We accounted for external factors that reduce infectious case fatality and diminish the vaccine-attributable impact on mortality. We did not include the downstream benefit of vaccination on non-communicable disease mortality (eg, of diarrhoea on malnutrition), nor broader economic benefit or community development gains that vaccination might facilitate, since the magnitude of causal attribution is more difficult to quantify.^30^ We also did not include possible heterologous effects of vaccines on epitopically non-specific immune training or other potential mechanisms. Such effects might mean that we underestimated the benefits of some vaccines (eg, BCG and measles) or did not sufficiently discount the benefits of others (eg, DTP-containing vaccines). The methods used in this study are well suited for a more thorough assessment of the possible population impacts of potential heterologous effects, but this is beyond the current scope. We cannot claim a complete analysis of the impact of immunisation, since we exclude vaccines such as those against COVID-19, which is arguably yet to achieve equilibrium; influenza, which is subject to local-level variation in seasonality and immunity profiles; and HPV, a vaccination programme which can anticipate a rapid increase in impact in the coming years. We did not include vaccines used for outbreaks such as cholera or Ebola; vaccines targeting disease occurring in adult life; or those used largely in high-income settings such as varicella, herpes zoster, or mumps, and the counterfactual assumed a smallpox free world, meaning that we did not account for the enormous benefit achieved by its eradication. The risk of double counting is a limitation, but we have shown that this has only a small effect on our estimates. We have presented global and regional findings, which delimits the geographical resolution at which conclusions can be drawn. Ongoing work to extend these models in consultation with member states is underway. The calendar-year impact of vaccination over the last 50 years was captured; compared with birth cohort-based or year-of-vaccination approaches, which require longer term projection based on broad assumptions, the calendar-year-based approach does not fully account for any post-2024 lifetime vaccination impacts, especially for diseases that occur later in life, implying a substantial underestimate for diseases such as hepatitis B.^31^ For the above reason, HPV, first licensed in 2006 and introduced more widely in the 2010s, was excluded from the analysis due to incomparability of timeframe.

The modelling used in this study were similar to previously conducted estimates against previously conducted estimates that were restricted in time and space. Other estimates projecting future impact as part of the Immunization Agenda 2030 that include the high coverage targets for the HPV vaccine have suggested that an even greater number of annual deaths averted over the life course are achievable.^18^, ^23^, ^32^ This imperative is highly dependent on achieving post-COVID-19 pandemic recovery and restoration of the trajectory to Immunization Agenda 2030 targets; achieving and maintaining universally high coverage with measles-containing vaccines (a principle aim of the Big Catch-up initiative; panel); the introductions of the much-anticipated malaria, respiratory syncytial virus, and other potential high impact vaccines; and achieving universal high coverage with an HPV vaccine (a must-win for Gavi, The Vaccine Alliance). HPV vaccine coverage is currently reaching only 21% of adolescent girls globally and is still far from the coverage targets of the WHO Cervical Cancer Elimination strategy, which aims to achieve HPV vaccination for 90% of all adolescent girls by 2030.^27^, ^33^

The first post-COVID-19 pandemic release of the WHO and UNICEF Estimates of National Immunization Coverage (known as WUENIC) showed that countries that had sustained improvements in vaccine coverage in the years before the pandemic also made more resilient recoveries from pandemic impacts on the programme than countries with plateaued vaccine coverage.^27^ The findings of this study make the related point that the remarkable achievements of vaccination are accumulated through consistent layered data-driven and operationally realistic efforts over years. Stakeholders need to protect the gains of EPI, sustain coverage, target remaining gaps, and think of immunisation programmes as the foundation of pandemic preparedness and of strong and resilient health systems. We are at an historic moment in infectious disease control. The large and ubiquitous gains that can be achieved have, through concerted collaborative effort, been achieved. The next 50 years of what has now become the Essential, rather than Expanded, Programme on Immunization, will require improvements in targeting and reach, especially for measles vaccines, amid future complex realities for unvaccinated and under-vaccinated children and communities. Continuous engagement of communities in vaccine uptake is crucial since hard won gains can so easily be lost. The next 50 years hold great promise, but need collective and sustained determination to deliver.

## Data sharing

All data sources and analytic code are available at https://github.com/WorldHealthOrganization/epi50-vaccine-impact. The entire repository can be downloaded at https://zenodo.org/records/10980462.

## Declaration of interests

CLT and KAMG assert that their employer, Imperial College, receives funding for the Vaccine Impact Modelling Consortium from the Bill & Melinda Gates Foundation; Gavi, the Vaccine Alliance; and the Wellcome Trust. CLT has received consulting fees from GSK for attending an advisory board meeting on CMV vaccines in May, 2022 and is pro bono Chair of the Scientific Advisory Panel of the Meningitis Research Foundation. HF asserts that her employer, London School of Hygiene & Tropical Medicine, receives funding for the Vaccine Impact Modelling Consortium from the Gates Foundation. JFM asserts that his employer, University of Washington, receives grant funding from Gavi and from the Gates Foundation. KB and KMT assert that their organisation Kid Risk holds a cooperative agreement with the US Centers for Disease Control and Prevention and holds grants from the Gates Foundation. MJF asserts that his employer, Penn State University, is a subrecipient of funds from Imperial College London for a grant from Gavi and that he holds grants from the Gates Foundation and the US National Science Foundation. MJ asserts that his employer, London School of Hygiene & Tropical Medicine, receives funding from the UK National Institute of Health Research, RCUK; the Gates Foundation; Gavi; the Wellcome Trust; WHO; the European Commission; the US Centers for Disease Control and Prevention; the Hong Kong Special Administrative Region Government; and the Task Force for Global Health. RAH and SPS assert that their employer, University of Cape Town, receives grant funding from the African Field Epidemiology Network and the US Centers for Disease Control and Prevention. RGW asserts that he receives funding from the Wellcome Trust (grant numbers 218261/Z/19/Z), National Institutes of Health (1R01AI147321-01, G-202303-69963, and R-202309-71190), European and Developing Countries Clinical Trials Partnership (RIA208D-2505B), UK Medical Research Council (CCF17-7779 via SET Bloomsbury), UK Economic and Social Research Council (ES/P008011/1), Bill & Melinda Gates Foundation (INV-004737 and INV-035506), and WHO (2020/985800-0). AL, KLO-B, NB-Z, PL, RCWH, and SYS work for WHO. All other authors declare no competing interests.

## References

[1] World Health Assembly (1974). WHO expanded programme on immunization. https://iris.who.int/handle/10665/92778.

[2] Chan M (2014). The contribution of immunization: saving millions of lives, and more. Public Health Rep.

[3] Cornejo S, Chevez A, Ozturk M (2023). [The Pan American Health Organization's Revolving Fund for access to vaccines: 43 years responding to the regional immunization programO Fundo Rotativo para Acesso a Vacinas da Organização Pan-Americana da Saúde: 43 anos respondendo ao Programa Regional de Imunizações]. Rev Panam Salud Publica.

[4] UNICEF (2018). Moving with the times: 1980–1988—discover the importance of data and research in UNICEF's efforts for child survival and development. https://www.unicef.org/stories/learning-experience-19801988.

[5] O'Brien K (2024).

[6] Institute of Medicine (1993).

[7] WHO Strategic Advisory Group of Experts (SAGE) on Immunization. https://www.who.int/groups/strategic-advisory-group-of-experts-on-immunization/about.

[8] Gavi, the Vaccine Alliance (2023). A history of Gavi, the Vaccine Alliance. https://www.youtube.com/watch?v=7QTf5CPC1Tw.

[9] Gavi, the Vaccine Alliance (2019). ADIPs and Hib Initiative evaluation. https://www.gavi.org/our-impact/evaluation-studies/adips-and-hib-initiative-evaluation.

[10] Gavi, the Vaccine Alliance (2021). Pneumococcal AMC. https://www.gavi.org/investing-gavi/innovative-financing/pneumococcal-amc.

[11] PATH (2015). The Meningitis Vaccine Project: a groundbreaking partnership. https://www.path.org/our-impact/articles/about-meningitis-vaccine-project/.

[12] WHO Malaria vaccine implementation programme. https://www.who.int/initiatives/malaria-vaccine-implementation-programme.

[13] WHO (2024). The Access to COVID-19 Tools (ACT) Accelerator. https://www.who.int/initiatives/act-accelerator.

[14] WHO, Gavi, The Vaccine Alliance, Immunization Agenda 2030, UNICEF (2023).

[15] WHO (2023). WHO/UNICEF estimates of national immunization coverage. https://www.who.int/teams/immunization-vaccines-and-biologicals/immunization-analysis-and-insights/global-monitoring/immunization-coverage/who-unicef-estimates-of-national-immunization-coverage.

[16] WHO (2023). WHO immunization data portal. https://immunizationdata.who.int/.

[17] WHO WHO/IVB Supplementary Immunization Activities Database. https://immunizationdata.who.int/.

[18] Toor J, Echeverria-Londono S, Li X (2021). Lives saved with vaccination for 10 pathogens across 12 countries in a pre-COVID-19 world. eLife.

[19] United Nations Department of Economic and Social Affairs Population Division (2022). The 2022 Revision of World Population Prospects. https://population.un.org/wpp/.

[20] Eilertson KE, Fricks J, Ferrari MJ (2019). Estimation and prediction for a mechanistic model of measles transmission using particle filtering and maximum likelihood estimation. Stat Med.

[21] Verguet S, Johri M, Morris SK, Gauvreau CL, Jha P, Jit M (2015). Controlling measles using supplemental immunization activities: a mathematical model to inform optimal policy. Vaccine.

[22] Badizadegan K, Kalkowska DA, Thompson KM (2022). Polio by the numbers—a global perspective. J Infect Dis.

[23] Carter A, Msemburi W, Sim SY (2023). Modeling the impact of vaccination for the Immunization Agenda 2030: deaths averted due to vaccination against 14 pathogens in 194 countries from 2021 to 2030. Vaccine.

[24] Institute for Health Metrics and Evaluation Global Burden of Disease (GBD). https://www.healthdata.org/research-analysis/gbd.

[25] WHO Immunization and vaccines related implementation research advisory committee (IVIR-AC). https://www.who.int/groups/immunization-and-vaccines-related-implementation-research-advisory-committee.

[26] WHO (2020). Immunization Agenda 2030: a global strategy to leave no one behind. https://www.who.int/publications/m/item/immunization-agenda-2030-a-global-strategy-to-leave-no-one-behind.

[27] WHO (2023). Progress and challenges with achieving universal immunization coverage. https://www.who.int/publications/m/item/progress-and-challenges.

[28] WHO (2018). BCG vaccines: WHO position paper—February 2018. https://www.who.int/publications/i/item/who-wer9308-73-96.

[29] Clark RA, Mukandavire C, Portnoy A (2023). The impact of alternative delivery strategies for novel tuberculosis vaccines in low-income and middle-income countries: a modelling study. Lancet Glob Health.

[30] Jit M, Hutubessy R, Png ME (2015). The broader economic impact of vaccination: reviewing and appraising the strength of evidence. BMC Med.

[31] Echeverria-Londono S, Li X, Toor J (2021). How can the public health impact of vaccination be estimated?. BMC Public Health.

[32] Brisson M, Kim JJ, Canfell K (2020). Impact of HPV vaccination and cervical screening on cervical cancer elimination: a comparative modelling analysis in 78 low-income and lower-middle-income countries. Lancet.

[33] WHO Cervical Cancer Elimination Initiative. https://www.who.int/initiatives/cervical-cancer-elimination-initiative#cms.

