# Supplementary material for: Contribution of vaccination to improved survival and health: modelling 50 years of the Expanded Programme on Immunization
Source: Lancet. 2024 May 25;403(10441):2307–16. doi: 10.1016/S0140-6736(24)00850-X (PMC11140691; doi:10.1016/S0140-6736(24)00850-X)
Supplement: Supplementary appendix [file mmc1.pdf]

THE LANCET

Supplementary appendix

This appendix formed part of the original submission and has been peer reviewed. We post it as supplied by the authors.

Supplement to: Shattock AJ, Johnson HC, Sim SY, et al. Contribution of vaccination to improved survival and health: modelling 50 years of the Expanded Programme on Immunization. *Lancet* 2024; published online May 2. https://doi.org/10.1016/S0140-6736(24)00850-X.

Supplementary Information

Contents

Supplementary results....2
Historical impact by WHO region and income status....6
Contribution of vaccination to decrease in infant mortality by region....8
Temporal impact by disease....9
Supplementary methods....11
Categorization of vaccine impact estimation....11
Vaccination coverage assumptions....14
Concept of disease-vaccine-activity....14
Definition of a 'Fully Vaccinated Person'....16
Vaccination coverage estimates....18
Relationship between routine and supplementary coverage....19
Coverage assumptions over the period 1974-1979....19
Switch between whole-cell and acellular pertussis vaccines....20
Transmission models (forms 1 and 2)....21
Static models (form 3)....24
Effective vaccine coverage....24
Maternal immunity assumptions....26
Model formulation....27
Extension of model estimates....33
Geographical imputation....33
Non-linear impact functions and temporal extrapolation....38
Uncertainty of estimates....42
Accounting for potential double counting....43
Code library and reproducibility....44
Supplementary References....45

Supplementary results

The following results complement those presented and discussed in the main manuscript. All results and figures can be reproduced by running the code associated with this analysis. See the *code library and reproducibility* section for details. Table S1 provides a complete disease breakdown of the results presented in Figure 1 of the main manuscript, including uncertainty bounds, which represent 95% credible intervals generated from 100 samples of Monte Carlo Markov Chain posteriors from impact function fits. See also the *uncertainty of estimates* section.

Figure S1 presents the results shown in Figure 1 of the main manuscripts disaggregated by WHO region. Figure S2 shows the equivalent disaggregated by World Bank income status. Note that for consistency of results, we selected the World Bank classification in 2024 for this figure.

Table S1 *Total 1974-2024 deaths averted and years of full health gained by disease, globally and by region. All values rounded to the nearest thousand.*

**Pathogen**	**Total deaths averted 1974-2024**		**Total YFH gained 1974-2024**	
	**All ages**	**Under 5s**	**All ages**	**Under 5s**
**Global**				
Diphtheria	353,000 [325,000 - 375,000]	319,000 [294,000 - 339,000]	24,713,000 [23,615,000 - 25,634,000]	22,345,000 [21,356,000 - 23,175,000]
Haemophilus influenzae type B	2,858,000 [2,811,000 - 2,892,000]	2,858,000 [2,811,000 - 2,892,000]	180,582,000 [177,932,000 - 181,781,000]	180,582,000 [177,932,000 - 181,781,000]
Hepatitis B	461,000 [444,000 - 479,000]	360,000 [346,000 - 374,000]	58,955,000 [57,246,000 - 60,746,000]	45,886,000 [44,555,000 - 47,280,000]
Japanese encephalitis	30,000 [29,000 - 31,000]	21,000 [20,000 - 21,000]	3,099,000 [2,984,000 - 3,166,000]	2,134,000 [2,055,000 - 2,180,000]
Measles	93,712,000 [90,477,000 - 96,901,000]	92,240,000 [89,055,000 - 95,378,000]	5,754,556,000 [5,548,055,000 - 5,959,209,000]	5,664,118,000 [5,460,863,000 - 5,865,555,000]
Neisseria meningitidis A	2,000 [2,000 - 3,000]	1,000 [1,000 - 1,000]	193,000 [177,000 - 203,000]	104,000 [96,000 - 110,000]
Pertussis	13,155,000 [12,587,000 - 13,586,000]	11,638,000 [11,163,000 - 11,998,000]	1,042,361,000 [984,322,000 - 1,086,420,000]	918,646,000 [868,741,000 - 956,544,000]
Poliomyelitis	1,570,000 [1,570,000 - 1,570,000]	888,000 [888,000 - 888,000]	755,344,000 [755,344,000 - 755,344,000]	427,160,000 [427,160,000 - 427,160,000]
Rotavirus	395,000 [388,000 - 402,000]	395,000 [388,000 - 402,000]	22,352,000 [21,926,000 - 22,655,000]	22,352,000 [21,926,000 - 22,655,000]
Rubella	285,000 [258,000 - 307,000]	0 [0 - 0]	21,265,000 [19,212,000 - 23,117,000]	0 [0 - 0]
Streptococcus pneumoniae	1,623,000 [1,600,000 - 1,638,000]	1,623,000 [1,600,000 - 1,638,000]	101,749,000 [99,958,000 - 102,553,000]	101,749,000 [99,958,000 - 102,553,000]
Tetanus	27,955,000 [26,991,000 - 28,745,000]	27,447,000 [26,502,000 - 28,221,000]	1,365,695,000 [1,298,894,000 - 1,414,916,000]	1,329,515,000 [1,264,519,000 - 1,377,271,000]
Tuberculosis	10,902,000 [10,658,000 - 11,126,000]	7,688,000 [7,516,000 - 7,846,000]	844,309,000 [761,502,000 - 903,650,000]	595,373,000 [536,981,000 - 637,218,000]
Yellow fever	557,000 [534,000 - 568,000]	305,000 [294,000 - 311,000]	31,135,000 [30,315,000 - 31,882,000]	18,002,000 [17,605,000 - 18,351,000]

**TOTAL**	153,858,000 [148,673,000 - 158,621,000]	145,782,000 [140,875,000 - 150,308,000]	10,206,307,000 [9,781,483,000 - 10,571,275,000]	9,327,967,000 [8,943,747,000 - 9,661,832,000]
**African region**				
Diphtheria	210,000 [203,000 - 216,000]	190,000 [184,000 - 195,000]	16,017,000 [15,346,000 - 16,580,000]	14,501,000 [13,894,000 - 15,010,000]
Haemophilus influenzae type B	1,536,000 [1,518,000 - 1,549,000]	1,536,000 [1,518,000 - 1,549,000]	95,557,000 [94,398,000 - 96,195,000]	95,557,000 [94,398,000 - 96,195,000]
Hepatitis B	128,000 [125,000 - 130,000]	99,000 [97,000 - 101,000]	16,302,000 [15,935,000 - 16,577,000]	12,658,000 [12,373,000 - 12,872,000]
Measles	28,660,000 [27,761,000 - 29,524,000]	28,210,000 [27,325,000 - 29,060,000]	1,510,601,000 [1,461,081,000 - 1,555,606,000]	1,486,860,000 [1,438,119,000 - 1,531,158,000]
Neisseria meningitidis A	2,000 [2,000 - 3,000]	1,000 [1,000 - 1,000]	192,000 [177,000 - 203,000]	104,000 [96,000 - 110,000]
Pertussis	4,191,000 [3,990,000 - 4,338,000]	3,805,000 [3,628,000 - 3,934,000]	342,809,000 [322,171,000 - 358,548,000]	310,648,000 [292,286,000 - 324,686,000]
Poliomyelitis	151,000 [151,000 - 151,000]	86,000 [86,000 - 86,000]	73,993,000 [73,993,000 - 73,993,000]	41,844,000 [41,844,000 - 41,844,000]
Rotavirus	228,000 [224,000 - 232,000]	228,000 [224,000 - 232,000]	12,838,000 [12,577,000 - 13,015,000]	12,838,000 [12,577,000 - 13,015,000]
Rubella	45,000 [35,000 - 54,000]	0 [0 - 0]	3,806,000 [2,988,000 - 4,551,000]	0 [0 - 0]
Streptococcus pneumoniae	1,028,000 [1,014,000 - 1,038,000]	1,028,000 [1,014,000 - 1,038,000]	65,288,000 [64,125,000 - 65,778,000]	65,288,000 [64,125,000 - 65,778,000]
Tetanus	9,545,000 [9,161,000 - 9,875,000]	9,487,000 [9,106,000 - 9,816,000]	424,369,000 [401,079,000 - 438,574,000]	420,085,000 [396,995,000 - 434,108,000]
Tuberculosis	6,568,000 [6,368,000 - 6,752,000]	4,631,000 [4,491,000 - 4,761,000]	519,116,000 [441,929,000 - 574,107,000]	366,060,000 [311,631,000 - 404,837,000]
Yellow fever	549,000 [526,000 - 560,000]	300,000 [288,000 - 305,000]	30,832,000 [30,024,000 - 31,568,000]	17,809,000 [17,421,000 - 18,150,000]
**TOTAL**	52,840,000 [51,078,000 - 54,420,000]	49,600,000 [47,961,000 - 51,077,000]	3,111,720,000 [2,935,824,000 - 3,245,295,000]	2,844,252,000 [2,695,759,000 - 2,957,764,000]
**Eastern Mediterranean region**				
Diphtheria	31,000 [28,000 - 35,000]	28,000 [26,000 - 32,000]	2,012,000 [1,877,000 - 2,128,000]	1,820,000 [1,698,000 - 1,925,000]
Haemophilus influenzae type B	433,000 [427,000 - 438,000]	433,000 [427,000 - 438,000]	28,987,000 [28,589,000 - 29,160,000]	28,987,000 [28,589,000 - 29,160,000]
Hepatitis B	25,000 [23,000 - 28,000]	19,000 [18,000 - 21,000]	3,034,000 [2,923,000 - 3,122,000]	2,359,000 [2,273,000 - 2,428,000]
Japanese encephalitis	0 [0 - 0]	0 [0 - 0]	7,000 [6,000 - 7,000]	5,000 [4,000 - 5,000]
Measles	12,887,000 [12,474,000 - 13,324,000]	12,685,000 [12,278,000 - 13,115,000]	796,715,000 [771,696,000 - 823,346,000]	784,194,000 [759,568,000 - 810,407,000]
Neisseria meningitidis A	0 [0 - 0]	0 [0 - 0]	1,000 [1,000 - 1,000]	0 [0 - 0]
Pertussis	1,871,000 [1,789,000 - 1,934,000]	1,570,000 [1,508,000 - 1,616,000]	144,347,000 [135,812,000 - 151,292,000]	120,133,000 [113,410,000 - 125,673,000]
Poliomyelitis	119,000 [119,000 - 119,000]	67,000 [67,000 - 67,000]	57,638,000 [57,638,000 - 57,638,000]	32,595,000 [32,595,000 - 32,595,000]
Rotavirus	63,000 [62,000 - 64,000]	63,000 [62,000 - 64,000]	3,822,000 [3,760,000 - 3,861,000]	3,822,000 [3,760,000 - 3,861,000]
Rubella	34,000 [30,000 - 37,000]	0 [0 - 0]	2,510,000 [2,240,000 - 2,762,000]	0 [0 - 0]

Streptococcus pneumoniae	288,000 [283,000 - 290,000]	288,000 [283,000 - 290,000]	19,111,000 [18,864,000 - 19,265,000]	19,111,000 [18,864,000 - 19,265,000]
Tetanus	8,501,000 [8,252,000 - 8,691,000]	8,432,000 [8,187,000 - 8,619,000]	409,269,000 [387,780,000 - 427,873,000]	404,152,000 [382,873,000 - 422,578,000]
Tuberculosis	822,000 [809,000 - 833,000]	580,000 [571,000 - 587,000]	61,605,000 [59,357,000 - 63,147,000]	43,441,000 [41,856,000 - 44,529,000]
Yellow fever	1,000 [1,000 - 1,000]	0 [0 - 0]	28,000 [28,000 - 28,000]	3,000 [3,000 - 3,000]
**TOTAL**	25,075,000 [24,296,000 - 25,793,000]	24,166,000 [23,426,000 - 24,850,000]	1,529,086,000 [1,470,570,000 - 1,583,631,000]	1,440,624,000 [1,385,494,000 - 1,492,431,000]
**European region**				
Diphtheria	2,000 [2,000 - 2,000]	2,000 [2,000 - 2,000]	146,000 [138,000 - 153,000]	132,000 [125,000 - 138,000]
Haemophilus influenzae type B	36,000 [36,000 - 37,000]	36,000 [36,000 - 37,000]	2,601,000 [2,578,000 - 2,622,000]	2,601,000 [2,578,000 - 2,622,000]
Hepatitis B	24,000 [22,000 - 27,000]	19,000 [17,000 - 21,000]	1,212,000 [1,174,000 - 1,242,000]	956,000 [925,000 - 980,000]
Measles	6,562,000 [6,251,000 - 6,855,000]	6,459,000 [6,152,000 - 6,747,000]	456,491,000 [435,741,000 - 477,387,000]	449,316,000 [428,893,000 - 469,885,000]
Pertussis	231,000 [218,000 - 242,000]	198,000 [187,000 - 207,000]	16,765,000 [15,823,000 - 17,462,000]	14,082,000 [13,323,000 - 14,653,000]
Poliomyelitis	244,000 [244,000 - 244,000]	138,000 [138,000 - 138,000]	116,329,000 [116,329,000 - 116,329,000]	65,786,000 [65,786,000 - 65,786,000]
Rotavirus	6,000 [6,000 - 6,000]	6,000 [6,000 - 6,000]	249,000 [246,000 - 251,000]	249,000 [246,000 - 251,000]
Rubella	14,000 [13,000 - 15,000]	0 [0 - 0]	1,070,000 [998,000 - 1,124,000]	0 [0 - 0]
Streptococcus pneumoniae	31,000 [30,000 - 32,000]	31,000 [30,000 - 32,000]	905,000 [884,000 - 917,000]	905,000 [884,000 - 917,000]
Tetanus	21,000 [16,000 - 25,000]	18,000 [14,000 - 21,000]	1,120,000 [924,000 - 1,280,000]	971,000 [801,000 - 1,109,000]
Tuberculosis	108,000 [106,000 - 110,000]	76,000 [75,000 - 77,000]	7,967,000 [7,853,000 - 8,068,000]	5,618,000 [5,538,000 - 5,689,000]
**TOTAL**	7,279,000 [6,944,000 - 7,594,000]	6,983,000 [6,657,000 - 7,289,000]	604,855,000 [582,688,000 - 626,836,000]	540,617,000 [519,099,000 - 562,031,000]
**Region of the Americas**				
Diphtheria	7,000 [4,000 - 10,000]	6,000 [4,000 - 9,000]	237,000 [203,000 - 269,000]	207,000 [180,000 - 233,000]
Haemophilus influenzae type B	181,000 [171,000 - 191,000]	181,000 [171,000 - 191,000]	4,858,000 [4,788,000 - 4,896,000]	4,858,000 [4,788,000 - 4,896,000]
Hepatitis B	18,000 [17,000 - 20,000]	15,000 [14,000 - 16,000]	309,000 [287,000 - 329,000]	243,000 [226,000 - 259,000]
Measles	14,894,000 [14,192,000 - 15,577,000]	14,660,000 [13,969,000 - 15,333,000]	1,018,337,000 [966,478,000 - 1,065,230,000]	1,002,333,000 [951,289,000 - 1,048,489,000]
Pertussis	368,000 [338,000 - 389,000]	311,000 [286,000 - 327,000]	28,788,000 [26,497,000 - 30,731,000]	23,953,000 [22,109,000 - 25,550,000]
Poliomyelitis	218,000 [218,000 - 218,000]	123,000 [123,000 - 123,000]	104,804,000 [104,804,000 - 104,804,000]	59,269,000 [59,269,000 - 59,269,000]
Rotavirus	31,000 [31,000 - 31,000]	31,000 [31,000 - 31,000]	963,000 [953,000 - 969,000]	963,000 [953,000 - 969,000]
Rubella	30,000 [28,000 - 31,000]	0 [0 - 0]	2,099,000 [1,968,000 - 2,192,000]	0 [0 - 0]

Streptococcus pneumoniae	69,000 [69,000 - 70,000]	69,000 [69,000 - 70,000]	2,326,000 [2,298,000 - 2,345,000]	2,326,000 [2,298,000 - 2,345,000]
Tetanus	65,000 [60,000 - 70,000]	63,000 [59,000 - 68,000]	2,698,000 [2,573,000 - 2,802,000]	2,615,000 [2,496,000 - 2,714,000]
Tuberculosis	231,000 [229,000 - 233,000]	163,000 [161,000 - 165,000]	14,239,000 [13,826,000 - 14,582,000]	10,040,000 [9,749,000 - 10,283,000]
Yellow fever	8,000 [8,000 - 8,000]	5,000 [5,000 - 6,000]	275,000 [262,000 - 285,000]	190,000 [181,000 - 197,000]
**TOTAL**	16,121,000 [15,363,000 - 16,849,000]	15,628,000 [14,891,000 - 16,338,000]	1,179,933,000 [1,124,937,000 - 1,229,436,000]	1,106,997,000 [1,053,538,000 - 1,155,203,000]
**South-East Asia region**				
Diphtheria	81,000 [78,000 - 85,000]	73,000 [71,000 - 77,000]	5,430,000 [5,295,000 - 5,552,000]	4,900,000 [4,777,000 - 5,010,000]
Haemophilus influenzae type B	601,000 [592,000 - 603,000]	601,000 [592,000 - 603,000]	43,365,000 [42,495,000 - 43,593,000]	43,365,000 [42,495,000 - 43,593,000]
Hepatitis B	73,000 [70,000 - 75,000]	57,000 [55,000 - 58,000]	10,486,000 [10,184,000 - 10,721,000]	8,158,000 [7,923,000 - 8,341,000]
Japanese encephalitis	20,000 [19,000 - 20,000]	14,000 [13,000 - 14,000]	2,151,000 [2,110,000 - 2,188,000]	1,481,000 [1,453,000 - 1,506,000]
Measles	20,517,000 [19,996,000 - 21,083,000]	20,194,000 [19,682,000 - 20,752,000]	1,264,122,000 [1,228,062,000 - 1,299,750,000]	1,244,255,000 [1,208,762,000 - 1,279,324,000]
Pertussis	4,428,000 [4,307,000 - 4,527,000]	3,910,000 [3,814,000 - 3,991,000]	366,568,000 [353,197,000 - 376,230,000]	323,138,000 [311,714,000 - 331,301,000]
Poliomyelitis	347,000 [347,000 - 347,000]	196,000 [196,000 - 196,000]	168,577,000 [168,577,000 - 168,577,000]	95,333,000 [95,333,000 - 95,333,000]
Rotavirus	62,000 [61,000 - 64,000]	62,000 [61,000 - 64,000]	4,150,000 [4,093,000 - 4,200,000]	4,150,000 [4,093,000 - 4,200,000]
Rubella	112,000 [105,000 - 117,000]	0 [0 - 0]	8,410,000 [7,891,000 - 8,955,000]	0 [0 - 0]
Streptococcus pneumoniae	137,000 [134,000 - 138,000]	137,000 [134,000 - 138,000]	10,091,000 [9,865,000 - 10,167,000]	10,091,000 [9,865,000 - 10,167,000]
Tetanus	9,237,000 [8,939,000 - 9,485,000]	8,883,000 [8,595,000 - 9,121,000]	503,183,000 [482,237,000 - 518,767,000]	477,844,000 [458,221,000 - 492,382,000]
Tuberculosis	2,416,000 [2,397,000 - 2,434,000]	1,704,000 [1,690,000 - 1,716,000]	189,866,000 [188,034,000 - 191,495,000]	133,886,000 [132,594,000 - 135,035,000]
**TOTAL**	38,030,000 [37,047,000 - 38,979,000]	35,830,000 [34,904,000 - 36,730,000]	2,576,398,000 [2,502,041,000 - 2,640,195,000]	2,346,599,000 [2,277,231,000 - 2,406,191,000]
**Western Pacific region**				
Diphtheria	21,000 [9,000 - 28,000]	19,000 [8,000 - 25,000]	871,000 [756,000 - 952,000]	785,000 [682,000 - 859,000]
Haemophilus influenzae type B	71,000 [68,000 - 73,000]	71,000 [68,000 - 73,000]	5,213,000 [5,084,000 - 5,314,000]	5,213,000 [5,084,000 - 5,314,000]
Hepatitis B	193,000 [187,000 - 199,000]	151,000 [146,000 - 155,000]	27,612,000 [26,744,000 - 28,754,000]	21,511,000 [20,834,000 - 22,400,000]
Japanese encephalitis	10,000 [10,000 - 10,000]	7,000 [7,000 - 7,000]	942,000 [868,000 - 970,000]	648,000 [598,000 - 668,000]
Measles	10,192,000 [9,803,000 - 10,537,000]	10,032,000 [9,649,000 - 10,372,000]	708,290,000 [684,998,000 - 737,889,000]	697,159,000 [674,233,000 - 726,293,000]
Pertussis	2,066,000 [1,946,000 - 2,155,000]	1,845,000 [1,739,000 - 1,923,000]	143,084,000 [130,823,000 - 152,157,000]	126,693,000 [115,899,000 - 134,682,000]
Poliomyelitis	491,000 [491,000 - 491,000]	278,000 [278,000 - 278,000]	234,002,000 [234,002,000 - 234,002,000]	132,332,000 [132,332,000 - 132,332,000]

Rotavirus	5,000 [5,000 - 6,000]	5,000 [5,000 - 6,000]	331,000 [296,000 - 359,000]	331,000 [296,000 - 359,000]
Rubella	50,000 [47,000 - 53,000]	0 [0 - 0]	3,370,000 [3,127,000 - 3,532,000]	0 [0 - 0]
Streptococcus pneumoniae	70,000 [70,000 - 71,000]	70,000 [70,000 - 71,000]	4,029,000 [3,922,000 - 4,081,000]	4,029,000 [3,922,000 - 4,081,000]
Tetanus	586,000 [563,000 - 600,000]	564,000 [542,000 - 576,000]	25,056,000 [24,301,000 - 25,621,000]	23,849,000 [23,134,000 - 24,380,000]
Tuberculosis	757,000 [749,000 - 764,000]	534,000 [528,000 - 538,000]	51,517,000 [50,503,000 - 52,250,000]	36,328,000 [35,613,000 - 36,845,000]
**TOTAL**	14,513,000 [13,945,000 - 14,987,000]	13,575,000 [13,037,000 - 14,024,000]	1,204,316,000 [1,165,424,000 - 1,245,882,000]	1,048,878,000 [1,012,627,000 - 1,088,212,000]

Historical impact by WHO region and income status

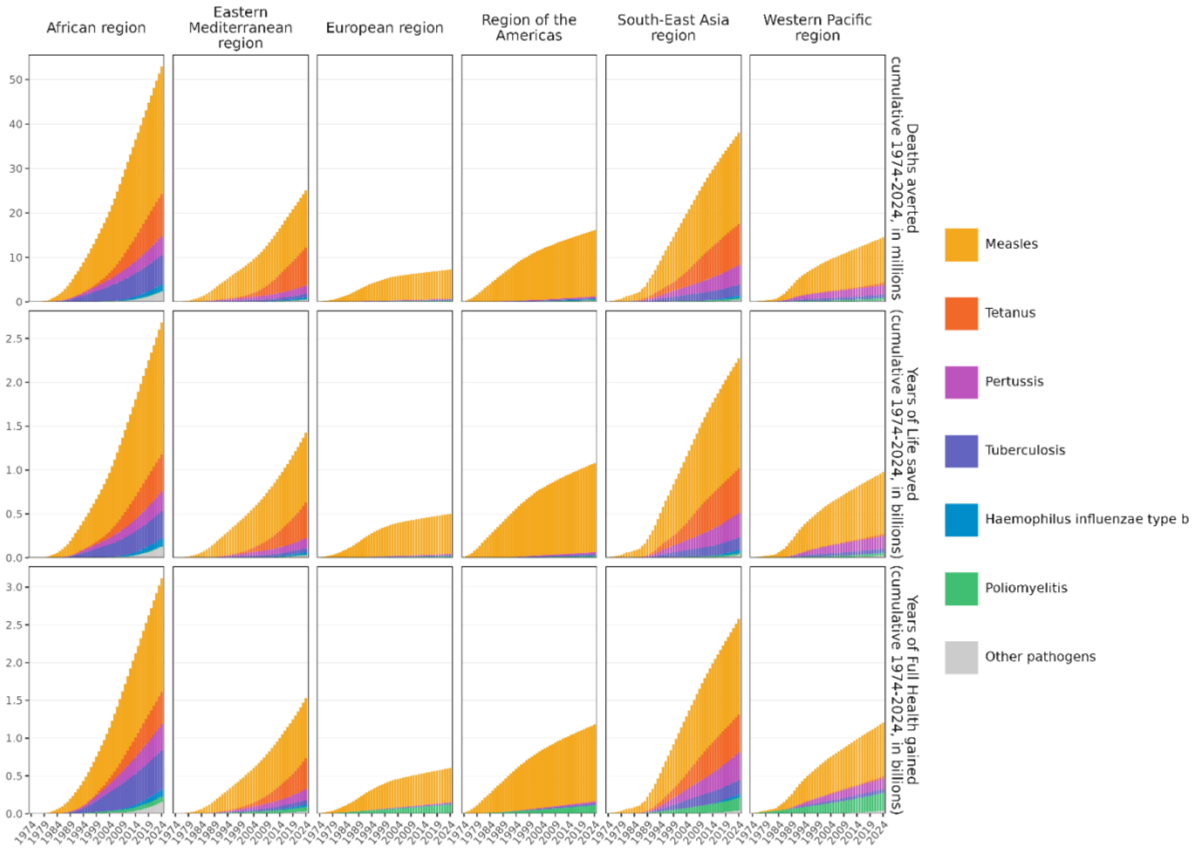

Figure S1 *Deaths averted, years of life saved, years of full health gained due to vaccination by WHO region.*

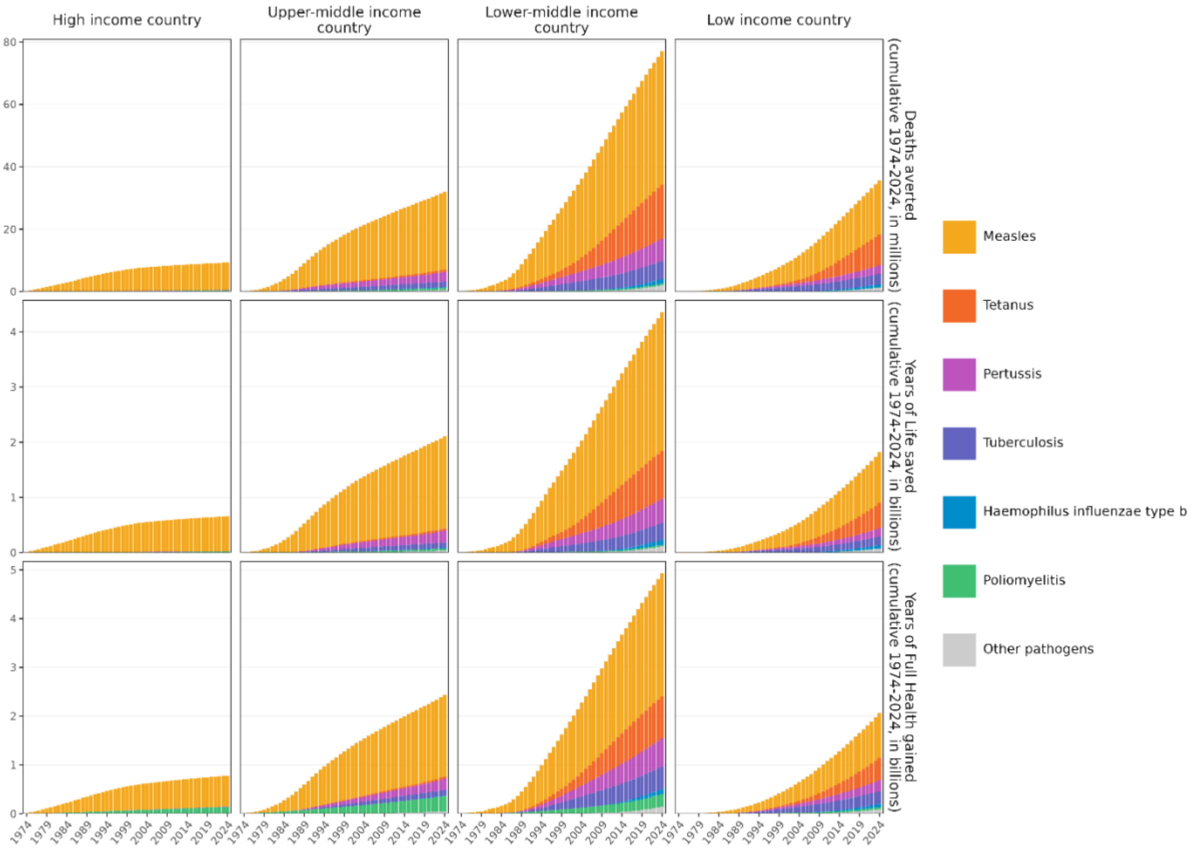

Figure S2 *Deaths averted, years of life saved, years of full health gained due to vaccination by World Bank income status (as classified in 2024).*

Contribution of vaccination to decrease in infant mortality by region

Figure S3 presents regional results corresponding to Figure 2 from the main manuscript. That is, the absolute all-cause decrease in infant mortality between 1974 and 2024 (yellow bars), the relative all-cause decrease (orange bars), and – crucially – the estimated contribution of vaccination to the decrease in infant mortality over the past 50 years (green bars). They grey bars represent the global totals, as presented in Figure 2 in the main manuscript. Of particular note, we found that over 50% of the considerable 50-year decrease in infant mortality in the African region is directly attributable to vaccination. That is, in the African region, vaccination has been the majority driver in increased infant survival over the past 50 years.

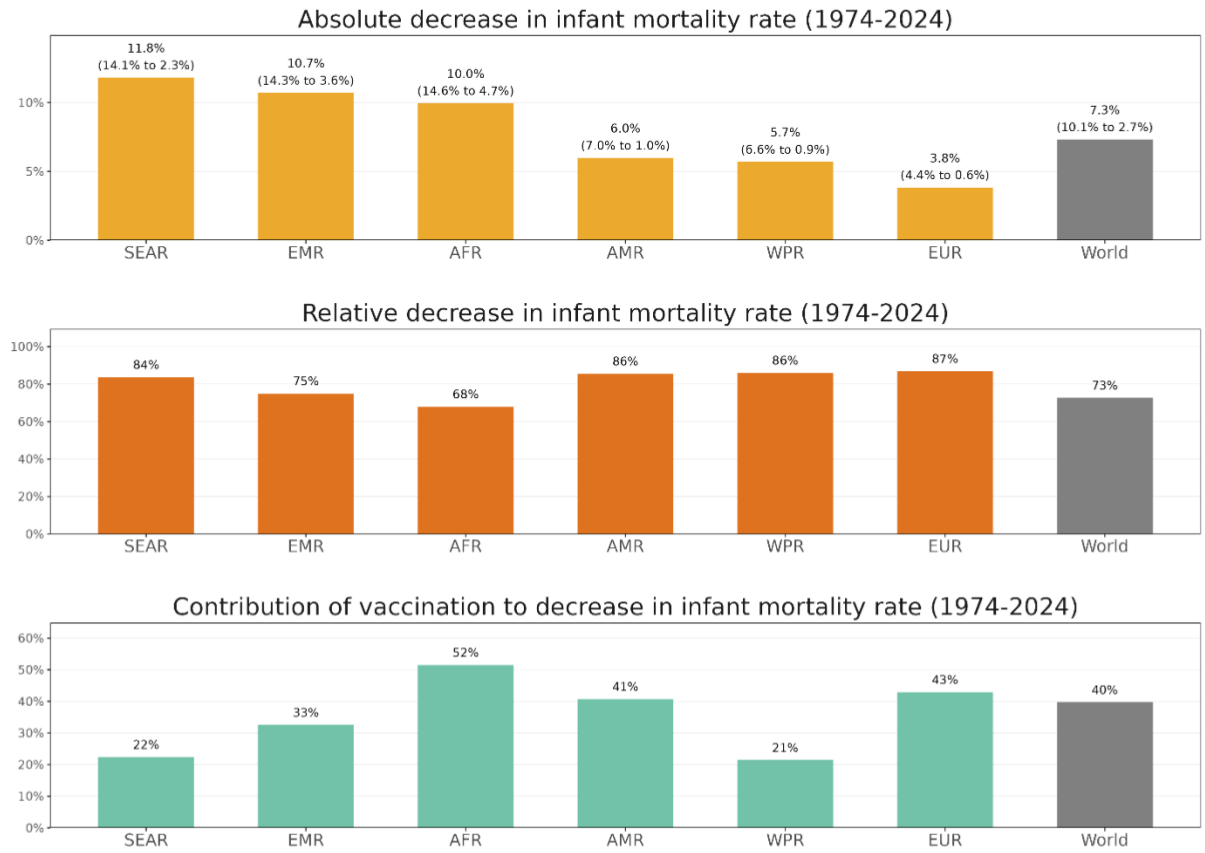

Figure S3 *Absolute and relative decrease in infant mortality and contribution of vaccination to the decrease in infant mortality, by region, 1974 – 2024. Regional acronyms: AFR = African region, AMR = Region of the Americas, EMR = Eastern Mediterranean region, EUR = European region, SEAR = South-East Asia region, WPR = Western Pacific region.*

Temporal impact by disease

Figure S4 illustrates the annual number of deaths averted for each of the 14 modelled pathogens by WHO region. The corresponding results for YFH gained are presented in Figure S5. Best estimates (solid lines) are presented with uncertainty bounds, which represent 95% credible intervals generated from 100 samples of Monte Carlo Markov Chain posteriors from impact function fits. See *uncertainty of estimates* section for further details of uncertainty estimation.

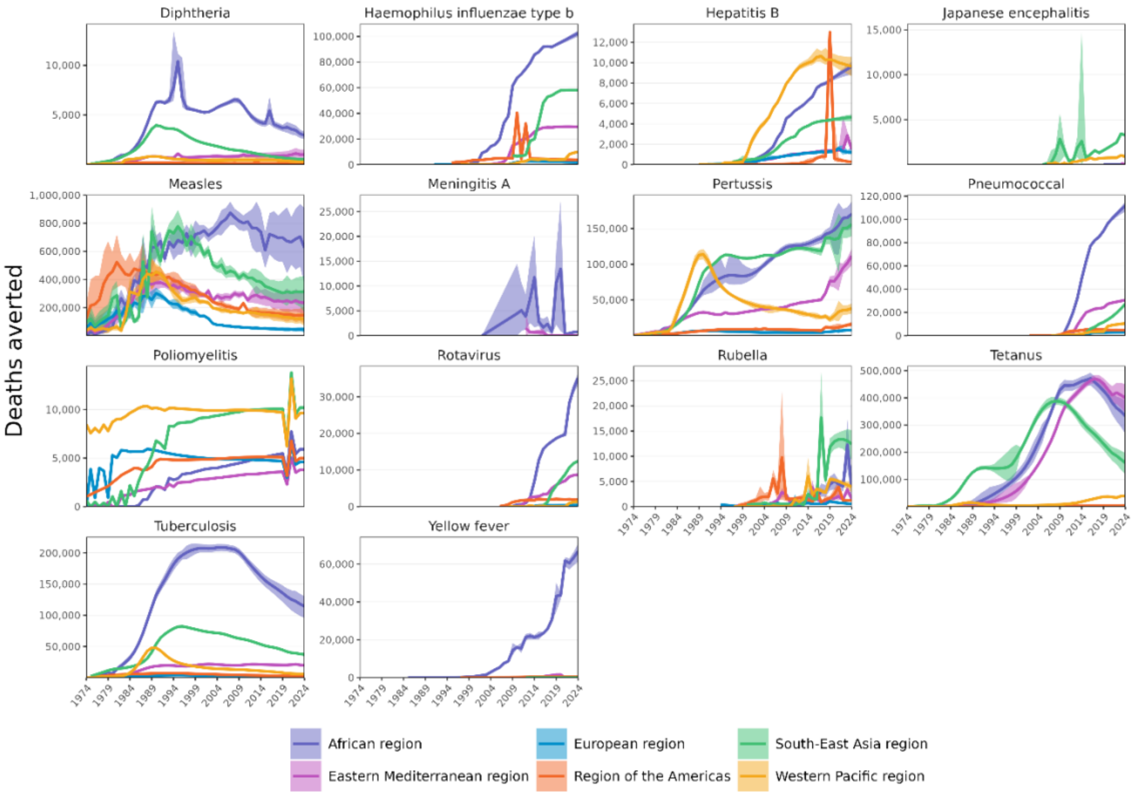

Figure S4 *Estimated number of deaths averted by each pathogen, in each WHO region.*

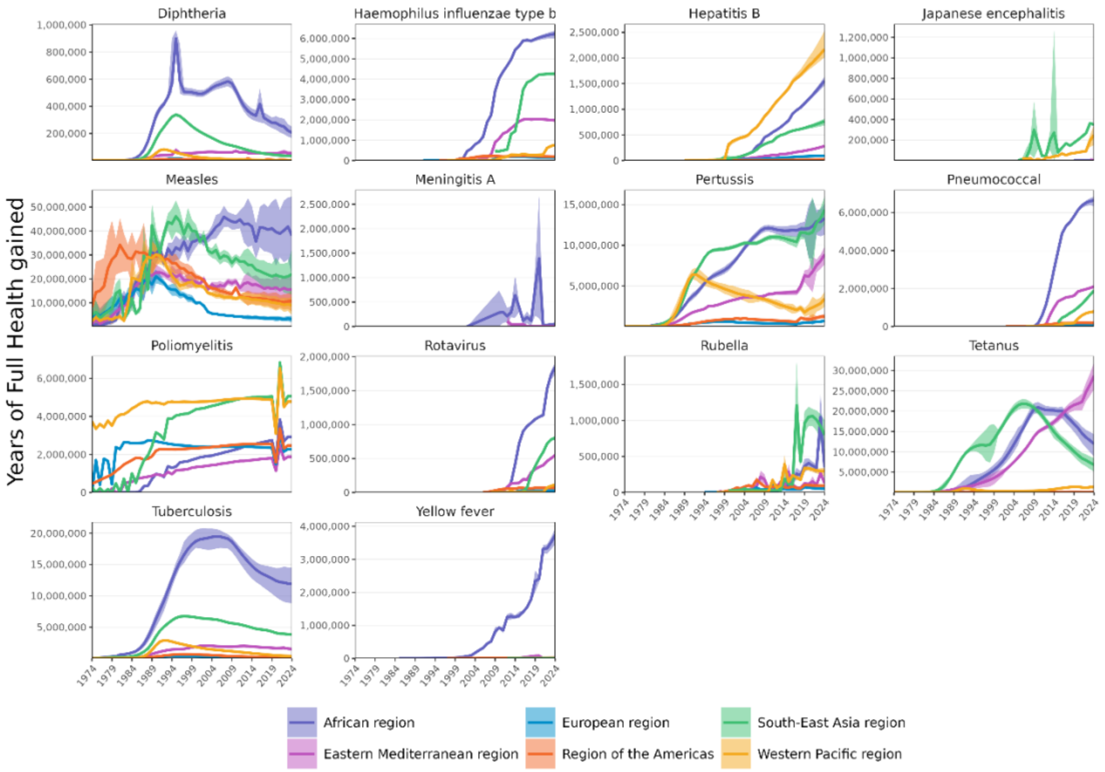

Figure S5 *Estimated number of years of full health gained by vaccination against each pathogen, in each WHO region.*

Supplementary methods

Categorization of vaccine impact estimation

The 14 pathogens considered in this analysis can be categorized into three mutually exclusive groups in terms of the techniques used to quantify vaccine impact (Table S2).

Table S2 *Approaches to quantifying vaccine impact.*

Form	Description	Pathogens
Form 1	Novel simulation of previously published transmission models.	Measles<br>Poliomyelitis
Form 2	Using outcomes from previously published transmission models in the Vaccine Impact Modelling Consortium (VIMC) portfolio.	*Haemophilus influenzae* type B<br>Hepatitis B<br>Japanese encephalitis<br>*Neisseria meningitidis* A<br>Rotavirus<br>Rubella<br>*Streptococcus pneumonia*<br>Yellow fever
Form 3	Using previously published static modelling approach built upon disease burden estimates from the Global Burden of Disease (GBD) study.	Diphtheria<br>Pertussis<br>Tetanus<br>Tuberculosis

In the case of forms 2 and 3, vaccine impact estimates were only available for a subset of the 1974-2024 analysis period. Further, in the case of form 2, vaccine impact estimates were only available for a subset of the 194 WHO Member States included in this study. Therefore, we used two separate extrapolation methods to estimate vaccination impact for the full 50-year global scope of this analysis:

i. Geographically, for Member States not included in the VIMC portfolio
ii. Temporally, to extend to dates for which values are not explicitly calculated through the above methods

These five sources (three forms and two extrapolations) of vaccine impact are described in detail in this supplement. Figure S6 illustrates the extent to which each of these impact estimation approaches are used in this analysis, in terms of the number of people vaccinated. Table S3 presents a summary in tabular form.

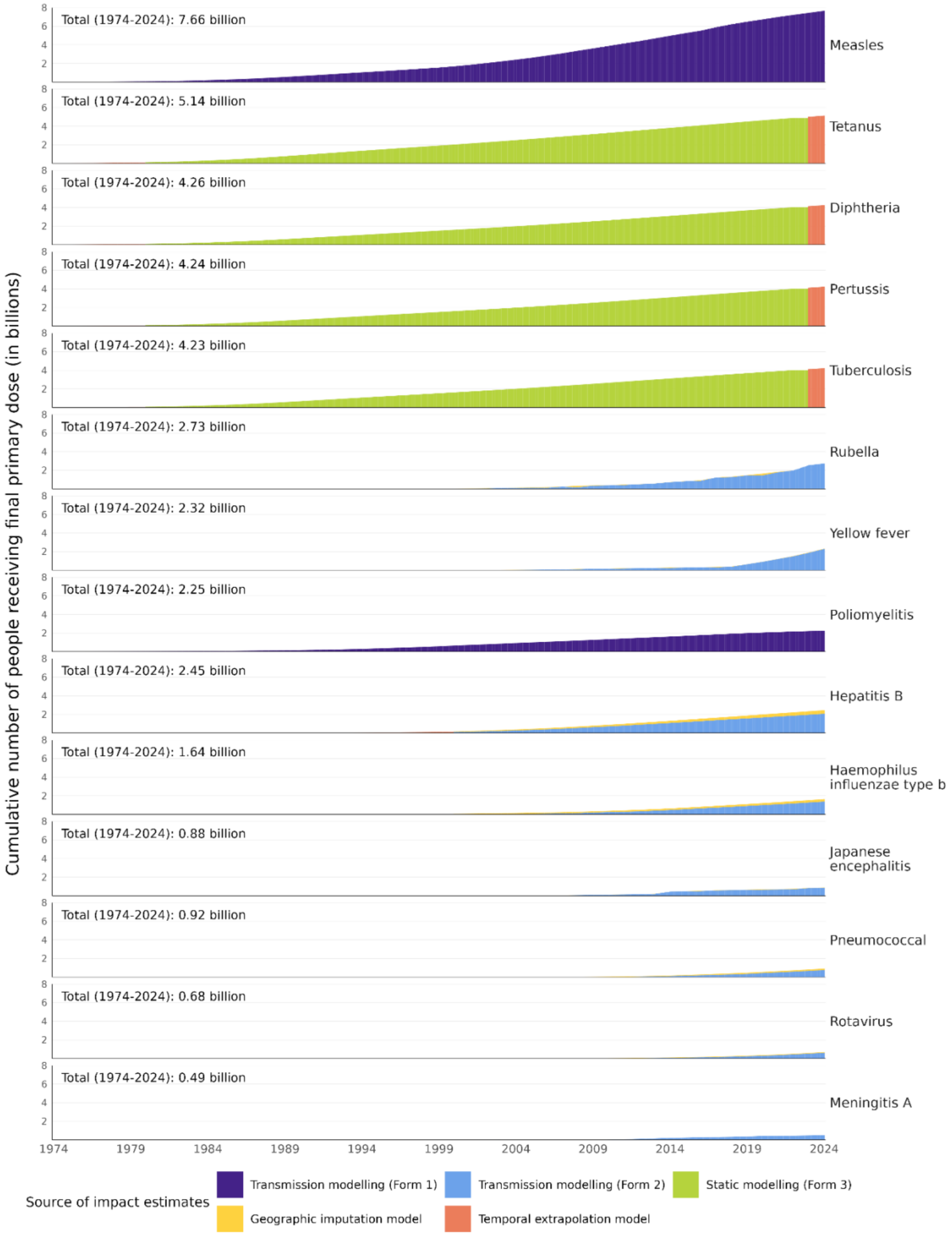

Figure S6 *The relative contribution of vaccine impact estimation methods.*

Table S3 Completeness of vaccine impact estimates, by form, geographical coverage and time.

Pathogen	Geographical scope	Of which requires geographical imputation	Temporal scope	Of which requires temporal extrapolation
Form 1				
Measles	194 Member States	None	1974-2024	None
Poliomyelitis	194 Member States	None	1974-2024	None
Form 2				
Haemophilus influenzae type B	194 Member States	84 Member States	1991-2024	1991-1999
Hepatitis B	192 Member States	82 Member States	1994-2024	1994-1999
Japanese encephalitis	23 Member States	5 Member States	2005-2024	None
Neisseria meningitidis A	26 Member States	None	2002-2024	None
Rotavirus	152 Member States	42 Member States	2006-2024	None
Rubella	133 Member States	23 Member States	1994-2024	1994-1999
Streptococcus pneumoniae	178 Member States	68 Member States	2002-2024	None
Yellow fever	69 Member States	36 Member States	1998-2024	1998-1999
Form 3				
Diphtheria	194 Member States	None	1974-2024	1974-1979<br>2022-2024
Pertussis	194 Member States	None	1974-2024	1974-1979<br>2022-2024
Tetanus	194 Member States	None	1974-2024	1974-1979<br>2022-2024
Tuberculosis	177 Member States	None	1974-2024	1974-1979<br>2022-2024

Vaccination coverage assumptions

Prior to describing each vaccine impact estimation method, we first define key concepts and present assumptions regarding vaccination coverage.

Concept of disease-vaccine-activity

Broadly, where evidence exists that different delivery modalities for a given vaccine may result in differing levels of per-dose impact, we attempt to capture such heterogeneity in the analysis. We do this through the concept of disease-vaccine activity. We provide a brief description of the various disease-vaccine-activities represented in this analysis here, and give further details where relevant in the following methodology sections.

For impact estimates derived from transmission models (forms 1 and 2), we consider routine and supplementary vaccine activities separately. In order to do this, we estimate impact attributable to each disease-vaccine activity by running full factorial of scenarios where routine and supplementary activities are introduced independently and synergistically, and then take the proportional difference of impact between these scenarios.

For impact estimates derived from static models (form 3), we make an assumption regarding how routine and supplementary activities contribute to an overall vaccination coverage and then model these activities together. However, primary and booster schedules are modelled separately (where appropriate), as are vaccine doses for pregnant women. Technical details are provided in the ‘static models’ section.

A complete list of all disease-vaccine-activities considered in this analysis are provided in Table S4.

Table S4 *Disease-vaccine-activities classifications.*

Disease	Vaccine	Activity
Forms 1 and 2		
Hepatitis B	Hepatitis B	Routine vaccination
	Hepatitis B birth dose	Routine vaccination
Haemophilus influenzae type B	*Haemophilus influenzae* type B	Routine vaccination
Japanese encephalitis	Japanese encephalitis	Routine vaccination
		Supplementary Immunization Activity
Measles	Measles-containing vaccine (dose 1)	Routine vaccination
	Measles-containing vaccine (dose 2)	Routine vaccination

	Measles-containing vaccine	Supplementary Immunization Activity
Neisseria meningitidis A	Meningitis A	Routine vaccination
		Supplementary Immunization Activity
Poliomyelitis	Inactivated polio vaccine	Routine and supplementary activities combined
	Oral polio vaccine	
Rotavirus	Rotavirus	Routine vaccination
Rubella	Rubella-containing vaccine	Routine and supplementary activities combined
Streptococcus pneumoniae	Pneumococcal vaccine	Routine vaccination
Yellow fever	Yellow fever	Routine vaccination
		Supplementary Immunization Activity
Form 3		
Diphtheria	Diphtheria-containing vaccine	Primary schedule
		Booster schedule
Pertussis	Whole cell pertussis-containing vaccine	Primary schedule
	Acellular pertussis-containing vaccine	Primary schedule
		Booster schedule
Tetanus	Tetanus-containing vaccine	Primary schedule
		Booster schedule
		Dose for pregnant women
Tuberculosis	Bacille Calmette-Guérin	Primary schedule

Definition of a 'Fully Vaccinated Person'

In this analysis we work with two complementary metrics relating to vaccine uptake: 1) vaccination coverage, and 2) Fully Vaccinated Persons (FVP). Vaccination coverage is defined in the classical sense. That is, the number of people vaccinated at some given point in time in some given population divided by the size of that population, resulting in a proportion between zero and one. FVP describes the total number of living people at some given point in time in some given population that have received a 'full' schedule of a given vaccine.

We stress here that FVP is a quantity defined for each disease-vaccine-activity independently. That is, there is no inter-vaccine dimension to this value. The number of doses required for an individual to be classified as a FVP for each disease-vaccine-activity in given in Table S5. In general, using FVPs only to quantify vaccine impact – and dismissing the potential impact of partially vaccinated people – could be considered a conservative approach. However, this assumption serves to offset the potential over-estimate of those receiving a specific dose of a vaccine; commonly an individual is considered to have received dose n if they receive a dose at the time dose n should be given, regardless of whether they have indeed received all prior doses. As such, the recorded coverage on dose n of a given vaccine can be considered an upper bound for the true value (assuming completeness of data).

We note here that an individual may be classified as a FVP for the primary schedule of a given vaccine e.g. diphtheria, but not for the corresponding booster schedule (Table S5). Thus, an individual receiving four doses of diphtheria-containing vaccine would be considered an FVP for diphtheria primary, but not for diphtheria booster. In the case of diphtheria (and also tetanus and pertussis), whilst this lack of consideration for an effect on a partial booster series may be considered conservative, this is offset by the assumption of sterile immunity following a full schedule of booster doses (see Figure S9).

The concept of FVP is used in this study primarily as a means in which to quantify cumulative effects of vaccine distribution. Vaccination coverage – being a proportion defined annually and bounded above by one – becomes meaningless in cumulative space over numerous years and is thus poorly equipped for such a use case. Consider a simple example of two consecutive years of 80% coverage for a cohort of 100 children for a single dose vaccine. The cumulative number of FVP in the case would be 160, which has a concrete meaning in our context. There is no meaningful equivalent for the vaccine coverage metric. Cumulative FVP are central concepts in both our temporal extrapolation and geographical imputation statistical models, described below.

Table S5 *Definitions of fully vaccinated people (FVP) by disease-vaccine-activity.*

Disease/vaccine	Scheduled doses	Notes
Diphtheria primary	3	
Diphtheria booster	3	Six doses required in total
Hepatitis B	3	
Hepatitis B birth dose	1	

Haemophilus influenzae type B	3	
Japanese encephalitis	2	
Measles: MCV1	1	Full temporal and geographical scope directly modelled, so FVP redundant concept for measles
Measles: MCV2	1	
Neisseria meningitidis A	1	
Pertussis primary (whole cell)	3	
Pertussis primary (acellular)	3	
Pertussis booster (acellular)	3	Six doses required in total
Poliomyelitis: IPV	4	Full temporal and geographical scope directly modelled, so FVP redundant concept for polio
Poliomyelitis: OPV	4	
Rotavirus	3	
Rubella	2	
Streptococcus pneumoniae	3	
Tetanus primary	3	
Tetanus booster	3	Six doses required in total
Tetanus pregnancy	1	
Tuberculosis: BCG	1	
Yellow fever	1	

Vaccination coverage estimates

We use four sources for vaccination coverage in this analysis:

1. WHO Immunization Information System (WIISE) database (for routine activities)
2. WHO Supplementary Immunization Activity (SIA) database
3. WHO Polio Information System (POLIS)
4. VIMC coverage estimates

We note here that VIMC coverage estimates are themselves a triangulation of multiple sources including WIISE and SIA databases, explained in detail by Toor et al [6] and illustrated in the VIMC dataviz tool. [36] For pathogens and Member States within the scope of VIMC, we use VIMC vaccination coverage estimates. For all pathogens and/or Member States outside the scope of VIMC, we calculate vaccination coverage using WIISE and SIA databases.

Figure S7 illustrates the contribution of each data source to overall coverage estimates by vaccine. The dashed line shows the total number of people (in billions) receiving a full course of vaccine within this activity. Figure S8 illustrates the age distribution of all data by source of coverage estimates. The wider, less targeted age range of SIA doses is prominent.

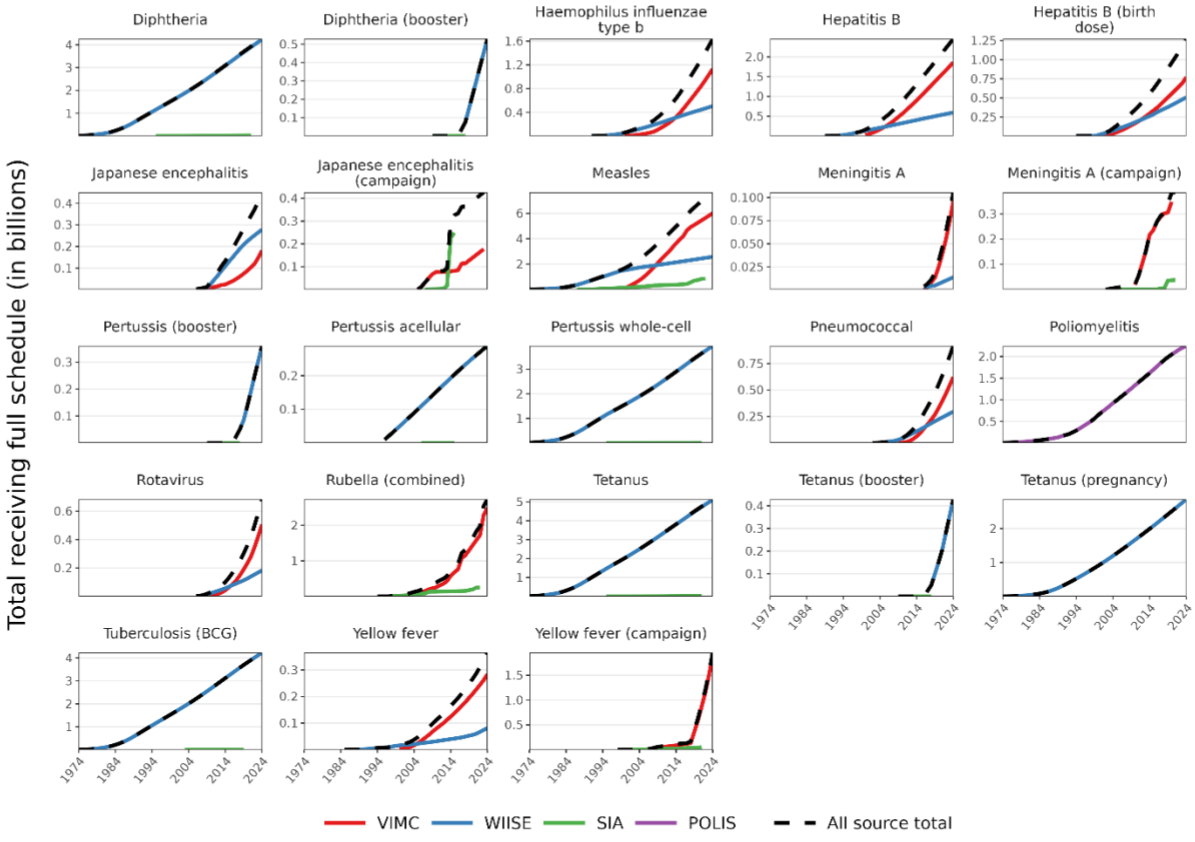

Figure S7 *Vaccination coverage by data source.*

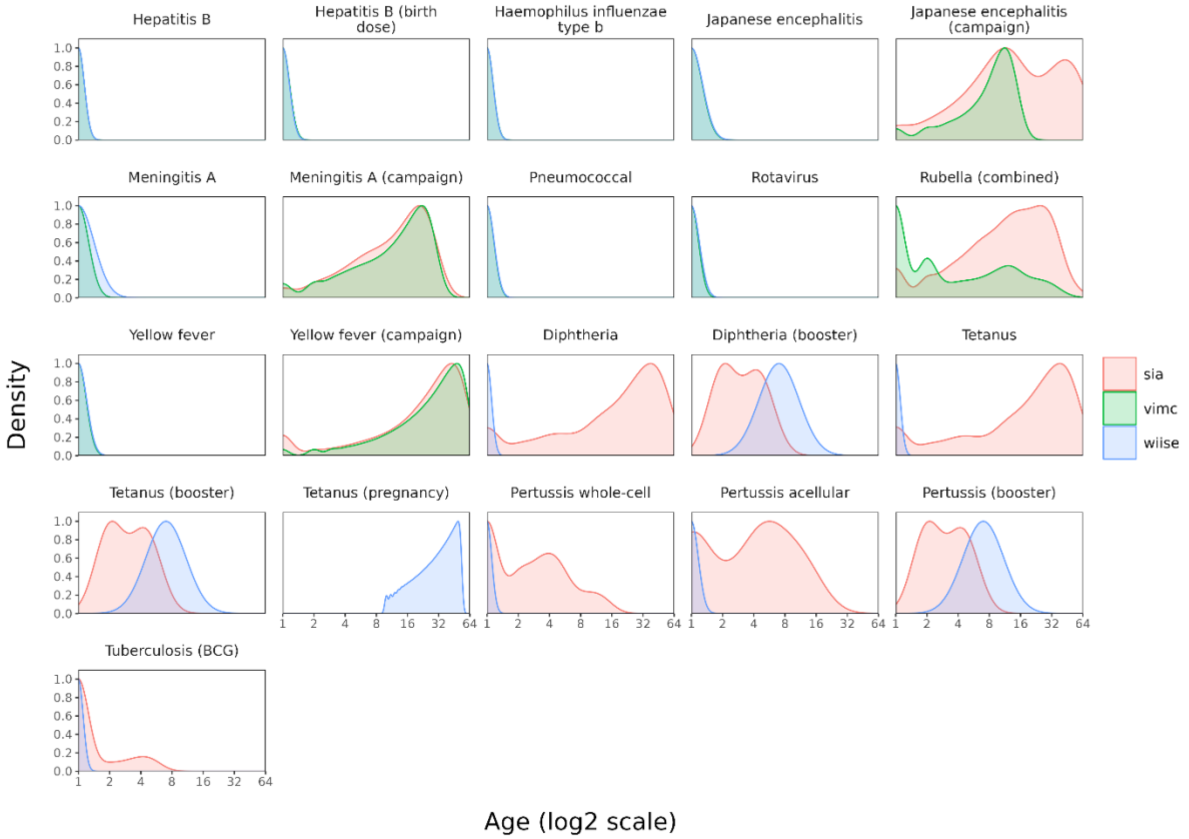

Figure S8 *Age distribution of vaccination, by data source.*

Relationship between routine and supplementary coverage

To derive an overall vaccination coverage estimate that incorporates both routine and supplementary coverage estimates (required for the static modelling approach (form 3)), we assume the synergistic effect follows the cumulative distribution function of the binomial distribution. That is, overall vaccination coverage, c, is defined as:

$$c_{k,y,a} = 1 - \left(1 - r_{k,y,a}\right)\left(1 - s_{k,y,a}\right)$$

for a given Member State k, year y, age group a, where r is coverage of routine vaccination, s is the coverage of supplementary activities. By using such a relationship, the underlying assumption is that supplementary doses are untargeted and therefore are proportionally likely to be received by an individual already vaccinated as they are by an unvaccinated individual. We note here that coverage remains capped at 100% by construction of this function.

Coverage assumptions over the period 1974-1979

Data is available for the period in 1980-2022 in the majority of cases, where applicable. For the 17 month post-data period we modelled (up to May 2024), we assumed vaccination coverage

was constant from 2022 levels. For the period 1974-1979, we used vaccination coverage in 1980 for the pathogens available at that time (DTP, BCG, measles, and polio vaccines) along with one of two assumptions:

1. Vaccination coverage was constant over the 1974-1980 period for Member States with **high-income** World Bank status in 1980.
2. Vaccination coverage linearly increased from zero over 1974-1980 period for Member States with **middle- or low-income** World Bank status in 1980.

We note here that assumption 2 is a conservative approach for estimating vaccine coverage in non-high-income Member States over this pre-1980 period. We also simulated the more ambitious assumption of constant coverage over 1974-1979 (set at 1980 levels) for middle- and low-income Member States, resulting in an additional 0.6% deaths averted (950,000 deaths) over this period. Figure S6 illustrates the general post-1980 scale up of vaccine coverage for available vaccines.

Switch between whole-cell and acellular pertussis vaccines

There are two formulations of pertussis vaccine in extant use, whole-cell (essentially killed, wP) and acellular (subunit, aP) pertussis vaccines. Acellular pertussis vaccine is generally better tolerated with lower reactogenicity. However in the acellular formulation clinical protection wanes faster, and susceptibility returns from about 10 years from vaccination. Since young adults may be susceptible, and pregnant women with low antibody titres provide little to no transplacental transfer to newborns, young infants born to susceptible families are at risk of infection and severe disease. wP is given in many low- and middle-income Member States as part of the Pentavalent vaccine (DwPT, Hib, HepB), though in March 2024 a wP hexavalent vaccine that includes injectable polio vaccine achieve prequalification. In many high-income Member States an aP hexavalent formulation is in use. The Strategic Advisory Group of Experts on Immunization position policy of 2015 reiterates standing policy and states: “National programmes may therefore consider the vaccination of pregnant women with 1 dose of TdaP in the 2nd or 3rd trimester and at least 15 days before the end of pregnancy where despite high infant coverage there would still be some infant mortality”. Although this is a global recommendation, in practice aP has been introduced mainly in high income Member States. To the best of our knowledge, Member States specific data on switch from wP to aP are not systematically available. We make the following assumptions in this analysis:

- Member States with **high-income** World Bank status in 1995 are assumed to have switched from wP to aP vaccines. From 1995 onwards, all pertussis vaccines in these Member States (primary and booster schedules) are assumed to be the acellular formulation.
- Member States with **middle- or low-income** World Bank status in 1995 are assumed to be using whole-cell pertussis vaccines for the entire analytical timeframe.
- We assume all booster doses given are of acellular formulation, regardless of Member State income status and formulation used for primary schedule.

Transmission models (forms 1 and 2)

For measles, we used the mean of two previously published models to estimate measles impact.[8,9,37] While these models are part of the VIMC portfolio, we ran a novel simulation with these two models using available data for historical measles coverage rate.

For polio, we collaborated with Kid Risk, Inc. who provided deaths, paralytic cases, and DALYs averted with the number of doses for OPV and IPV, building on a prior retrospective model that characterised the reduction in poliovirus cases due to historical poliovirus immunization through 2021 compared to the counterfactual scenario of no poliovirus vaccines.[10] The current analysis extends the model to account for poliovirus importation events and OPV and IPV vaccine delivery that occurred through mid 2024 For this analysis, the counterfactual scenario assumes no seasonality, but otherwise applies the same assumptions as previously reported.[10] To characterise mortality, the results conservatively assume fatality rates for paralytic polio cases based on the observed rates reported to the WHO and curated in WHO's polio information system POLIS. For the no vaccine scenario, this assumption assumes the development of sufficient global resources for respiratory support to maintain the observed vaccine scenario fatality rate during outbreak surges, which implies maintenance and expansion of polio wards in hospitals with iron lungs. In the absence of this capacity, greater fatality rates could have occurred during outbreaks than considered for the counterfactual scenario.

For eight pathogens, we used previously reported outcomes from 17 transmission models reporting to the Vaccine Impact Modelling Consortium (VIMC) (Table S6). The version of VIMC modelling estimates used in this study are associated with the identification number: 20240318-082736-d6c0daf9, as used in Toor et al.[6] These VIMC estimates are based upon short-term default coverage extrapolations from 2021 to mid-2024.

Table S6 *Models contributed by the Vaccine Impact Modelling Consortium (VIMC).*

Pathogen	Model name/institution	Model details
Hepatitis B	PRoGReSs, Centre for Disease Analysis Foundation[38]	A dynamic, deterministic disease burden model of hepatitis B virus transmission that calculates the annual prevalence, incidence, and mortality by stage of liver disease, serologic status (low-viral load, high-viral load, on-treatment), sex, and age.
	Imperial College London[39,40]	A dynamic, population-level, deterministic, transmission model structured by age, sex, and region. The model contains acute (Severe Acute and Non-severe Acute) and chronic (Immune Tolerant, Immune Reactive, Asymptomatic Carrier, Chronic Hepatitis B, Compensated Cirrhosis, Decompensated Cirrhosis and Liver Cancer) mutually exclusive disease states.

	Goldstein[41]	A static deterministic model that examines the mortality outcomes due to hepatitis B virus infection, including deaths of fulminant hepatitis, and deaths of liver cirrhosis and hepatocellular carcinoma as results of chronic hepatitis B.
Haemophilus influenzae type B	The Lives Saved Tool (LiST), Johns Hopkins University[42]	A static, deterministic, linear mathematical model for estimating the health impact of changes in coverage of 80 health interventions, including vaccines.<br><br>LiST generates estimates of Hib pneumonia and meningitis cases and deaths averted by the coverage scale-up of Hib vaccine.
	Universal Vaccine Decision Support model (UNIVAC), LSHTM[43]	A static cohort model with a finely disaggregated age structure (weeks of age <5 years, single years of age 5–99 years) that calculates impact (% reduction in cases, clinic visits, hospitalisations, lifelong sequelae, deaths and DALYs).<br><br>UNIVAC included estimates for non-severe Hib pneumonia, severe Hib pneumonia, Hib meningitis and Hib non-pneumonia/non-meningitis in children aged <5 years.
Japanese encephalitis	National University of Singapore[44]	A dynamic, deterministic model s a basic catalytic model for the force of infection. A 'bottom up' approach was used to generate deaths and cases i.e. from infection rates applying parameters governing the proportion of infections that are symptomatic and the proportion that die (case fatality ratio).
	University of Notre Dame[45,46]	A static, stochastic model of Japanese encephalitis virus transmission with a constant force of infection to calculate infections, cases and deaths.
*Measles**	DynaMICE, London School of Hygiene and Tropical Medicine (LSHTM) [9,37]	A dynamic, age-structured compartmental transmission model. Uses country-specific estimates for the basic reproduction number. Case fatality rates are defined as a function of incidence.
	Pennsylvania State University (PSU)[8]	A dynamic, age-structured, discrete time-step, annual SIR model which estimates the aggregate number of cases over one-year time steps.
	*While LSHTM and PSU models are part of the VIMC portfolio, we ran novel simulations with these two models for the purpose of this analysis.	
Neisseria meningitidis A	University of Cambridge[47]	A dynamic, compartmental transmission model of Neisseria meningitidis group A carriage and disease. Model developed for MenAfriVac.
	Kaiser Permanente Washington[48]	A dynamic, stochastic, age-structured, compartmental transmission model of *Neisseria meningitidis* A

Streptococcus pneumoniae	The Lives Saved Tool (LiST), Johns Hopkins University[42]	LiST generates estimates of pneumococcal pneumonia and meningitis cases and deaths averted by the coverage scale-up of pneumococcal conjugate vaccine.
	Universal Vaccine Decision Support model (UNIVAC), LSHTM[43]	UNIVAC included estimates for non-severe Pneumococcal pneumonia, severe Pneumococcal pneumonia, Pneumococcal meningitis, severe Pneumococcal non-pneumonia/non-meningitis in children aged <5 years, and cases of Pneumococcal acute otitis media.
Rotavirus	Emory University[49]	A dynamic, deterministic, age-structured compartmental transmission model that simulates rotavirus transmission and estimates disease incidence/burden in a given country.
	The Lives Saved Tool (LiST), Johns Hopkins University[42]	LiST generates estimates of rotavirus diarrhoea cases and deaths averted by the coverage scale-up of rotavirus vaccine.
	Universal Vaccine Decision Support model (UNIVAC), LSHTM[43]	UNIVAC includes estimates of rotavirus deaths <5 years and cases (non-severe and severe).
Rubella	Johns Hopkins University[50]	A dynamic, discrete-time, stochastic, age-structured, compartmental transmission model. A 'bottom up' approach was used to calculate congenital rubella syndrome cases, and deaths, from modeled output.
	Public Health England[51,52]	A dynamic, deterministic, age and sex-structured, compartmental, transmission model that calculates congenital rubella syndrome cases and deaths.
Yellow Fever	Imperial College London[53,54]	A static force of infection epidemiological model that uses published values of the proportion of infections which are severe and of case fatality rate to calculate the burden of disease.
	University of Notre Dame[53,55]	A static transmission model that assumes a constant force of infection for each endemic country. Scaled estimates were used to project the annual number of infections, and this quantity is multiplied by probabilities of disease and death.

More detailed model descriptions are available in VIMC's second consortium-wide paper.[6]

Static models (form 3)

Static models were developed to estimate vaccine impact for four diseases: diphtheria, tetanus, pertussis, and tuberculosis. A more basic formulation of these static models has been previously published.[11] Several enhancements to these static models have since been incorporated, and thus we fully derive these models here. The 2021 Global Burden of Disease estimates (GBD 2021) for four diseases were used for this analysis.[56-59] Broadly, vaccine impact – in terms of deaths averted and DALYs averted (or equivalently YHL gained) – is derived for each disease-vaccine-activity using three key quantities:

1. Disease-attributable mortality and morbidity as estimated by GBD [12]
2. Vaccine efficacy profiles
3. Vaccination coverage

Effective vaccine coverage

The last two quantities are combined to result in an estimate of 'effective vaccine coverage', which is then used to estimate disease-attributable mortality and morbidity in a hypothetical scenario of no historical vaccination. Vaccine impact is then calculated as the difference between the outcomes in this hypothetical 'no vaccine' scenario and the GBD burden estimates.

In this context, vaccine efficacy is interpreted as the reduction in probability of disease or death. Where appropriate, distinct vaccine efficacy profiles are derived for the primary schedule and any subsequent booster schedule, allowing for unique initial efficacy and waning immunity characterisations. For vaccines for pregnant women, a distinct efficacy profile is used for the effect on the newborn. In the case of pertussis, we derive distinct efficacy profiles for whole-cell and acellular vaccines.

The data used to inform each vaccine efficacy profile is detailed in Table S7. In most cases, data refer to initial efficacy (efficacy in the year of vaccination) or half-life (number of years taken for efficacy to decay to half of initial efficacy). Upon visual inspection of the data available, and following expert consultation, a functional form was assumed for each vaccine efficacy profile (Table S7). Generally, exponential decay functions were assumed for primary schedule and constant functions assumed for booster schedules, with the interpretation being that once an individual has received all doses in the primary and booster schedules (six doses for DTaP) immunity no longer decays. The BCG vaccine for TB was a special case, in which we assumed a sigmoidal decay. We fitted the parameters of the designated functional form using an Adaptive Stochastic Descent optimisation algorithm.[60] The optimisation process was repeated ten times for each disease-vaccine-activity using different initialisations to maximize the probability that the global optimum was identified. Table S7 states the fitted parameters for each pre-defined disease-vaccine-activity functional form. Figure S9 illustrates the resulting vaccine efficacy profiles along with the data used.

Table S7 Details of functional form used to represent vaccine efficacy – in terms of reduction in death or disease – for each statically modelled disease.

Disease	Vaccine	Activity	Efficacy data used [efficacy, years from vaccination]	Assumed functional form	Fitted parameters
Diphtheria	Diphtheria-containing vaccine	Primary schedule	*Initial efficacy:* [0.87, 0][61] [0.95, 0][62] *Half-life (of 87% initial efficacy):* [0.44, 19][63] [0.44, 27][64] *Half-life (of 95% initial efficacy):* [0.48, 19][63] [0.48, 27][64]	*Exponential decay* $y = ae^{-bt}$	$a = 0.90$ $b = 0.29$
		Booster schedule	*Booster efficacy:* [0.95, 0][65,66]	*Constant* $y = a$	$a = 0.95$
Pertussis	Whole cell pertussis-containing vaccine	Primary schedule	*Initial efficacy:* [0.94, 0][67] [0.92, 0][68] [0.99, 0][69] *15 year half life (expert opinion):* [0.46, 15]	*Exponential decay* $y = ae^{-bt}$	$a = 0.95$ $b = 0.046$
	Acellular pertussis-containing vaccine	Primary schedule	*Initial efficacy:* [0.84, 0][67] [0.80, 0][70] *75% efficacy drops 35% after 5 years:* [0.49, 5][71] *Vaccine efficacy of 41% after 8 years:* [0.41, 8][70]	*Exponential decay* $y = ae^{-bt}$	$a = 0.82$ $b = 0.093$
		Booster schedule	*Efficacy similar to primary series (expert opinion):* [0.80, 0][70]	*Constant* $y = a$	$a = 0.80$
Tetanus	Tetanus-containing vaccine	Primary schedule	*Initial efficacy:* [0.95, 0][72] *Half-life:* [0.48, 11][63] [0.48, 14][64]	*Exponential decay* $y = ae^{-bt}$	$a = 0.95$ $b = 0.054$
		Booster schedule	*Booster efficacy:* [0.95, 0][73]	*Constant* $y = a$	$a = 0.95$

		Pregnancy schedule	*Maternal immunity among neonates:* [0.45, 0][74] [0.65, 0][75] [0.94, 0][76]	*Step function* $y = \begin{cases} a, & t < 1 \\ 0, & t \geq 1 \end{cases}$	$a = 0.68$
Tuberculosis	Bacille Calmette-Guerin	Primary schedule	*Initial efficacy:* [0.66, 0][77] [0.66, 15][78] *Half-life of 20 years (expert opinion):* [0.33, 20] [0.10, 40]	*Sigmoidal decay* $y = a + \frac{b - a}{1 + \left(t/c\right)^{-d}}$	$a = 0.68$ $b = 0.097$ $c = 19.3$ $d = 10.0$

Maternal immunity assumptions

For vaccines delivered during pregnancy, we assume the effect on the pregnant women is equivalent to a booster dose. For the effect on the newborn, we use a distinct vaccine efficacy profile (Table S7 and Figure S9). The efficacy in the newborn is assumed to remain during the neo-natal phase (first 4 weeks of life) and then decay to zero. In effect, only neonates receive any benefit of maternal immunity, with no residual effect for post-neonatal infants.

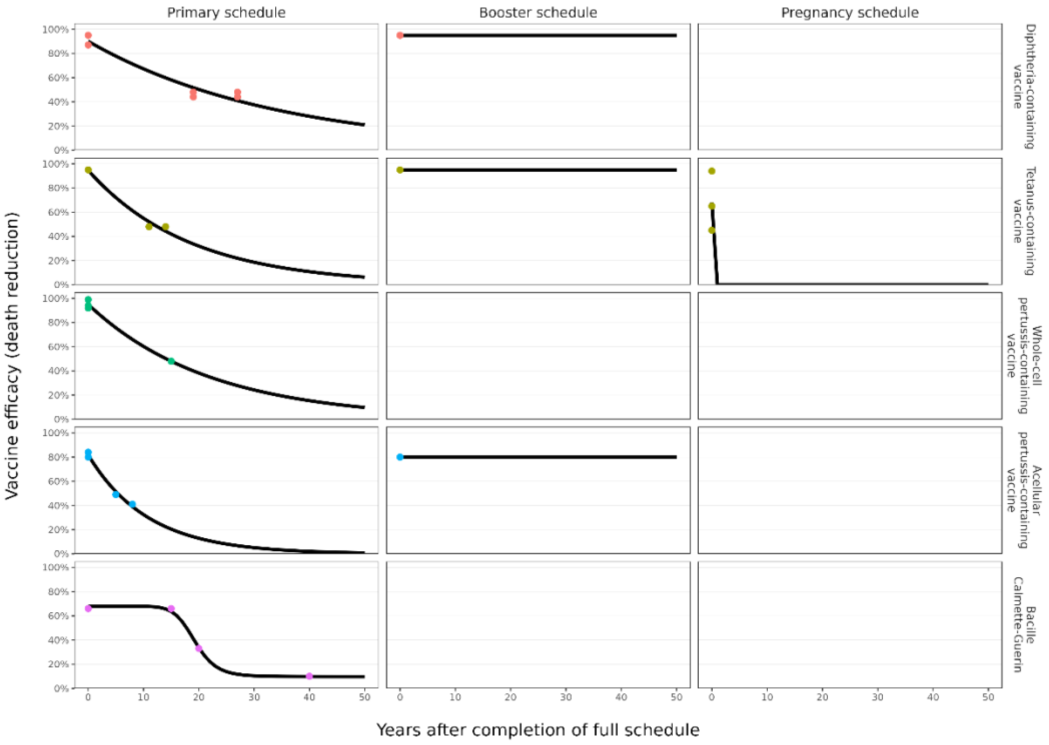

***Figure S9** Derived assumptions of effective protection of diphtheria, tetanus, pertussis and BCG vaccines against death, throughout the life course. Note that the efficacy associated with 'pregnancy schedule' is the effect on newborns, which decays to zero effect after the neonate phase of 28 days.*

Model formulation

For each disease-vaccine-activity x, we derive effective vaccine coverage e in each Member State k for each year y and single-year age group a as:

$$e_{x,k}(y,a) = 1 - \prod_{i=y-a}^{y} \left(1 - c_{x,k}(i, y-i+a) \cdot \varepsilon_x(y-i)\right)$$

Where $c_{x,k}(y,a)$ is vaccine coverage in year y for age a, and $\varepsilon_x(t)$ is vaccine efficacy t years after vaccination for the specific disease-vaccine-activity. Note that commonly only one term in this equation is non-trivial, given that routine vaccinations (which make up the vast majority of doses in the case of diphtheria, pertussis, tetanus, and tuberculosis) are generally targeted at a specific age group – commonly infants – each year.

It remains to aggregate effective vaccine coverage for a given disease. First, we aggregate effective vaccine coverage for each vaccine v that targets disease d, noting that two distinct vaccines are modelled for pertussis (whole cell and acellular). This process involves weighting between primary and booster schedule effect such that those receiving boosters are not double counted. Let disease-vaccine-activity $x = p_v$ represent the primary schedule for vaccine v, and $x = b_v$ the booster schedule, then we define:

$$e_{v,k}(y,a) = e_{p_v,k}(y,a)\left[1 - \frac{c_{b_v,k}(y,a)}{c_{p_v,k}(y,a)}\right] + e_{b_v,k}(y,a)$$

That is, effective coverage in the primary schedule is reduced by the proportion of primary FVP that are not booster FVP. We then aggregate up to effective vaccine coverage for disease d by considering the effects of each relevant vaccine to be additive. That is:

$$e_{d,k}(y,a) = \sum_{v} e_{v,k}(y,a)$$

Figure S10 illustrates global effective vaccine coverage of each vaccine in each year between 1974 and for age groups 0 to 50 years. Figure S11 represents the corresponding effective vaccine coverage summarised at disease level.

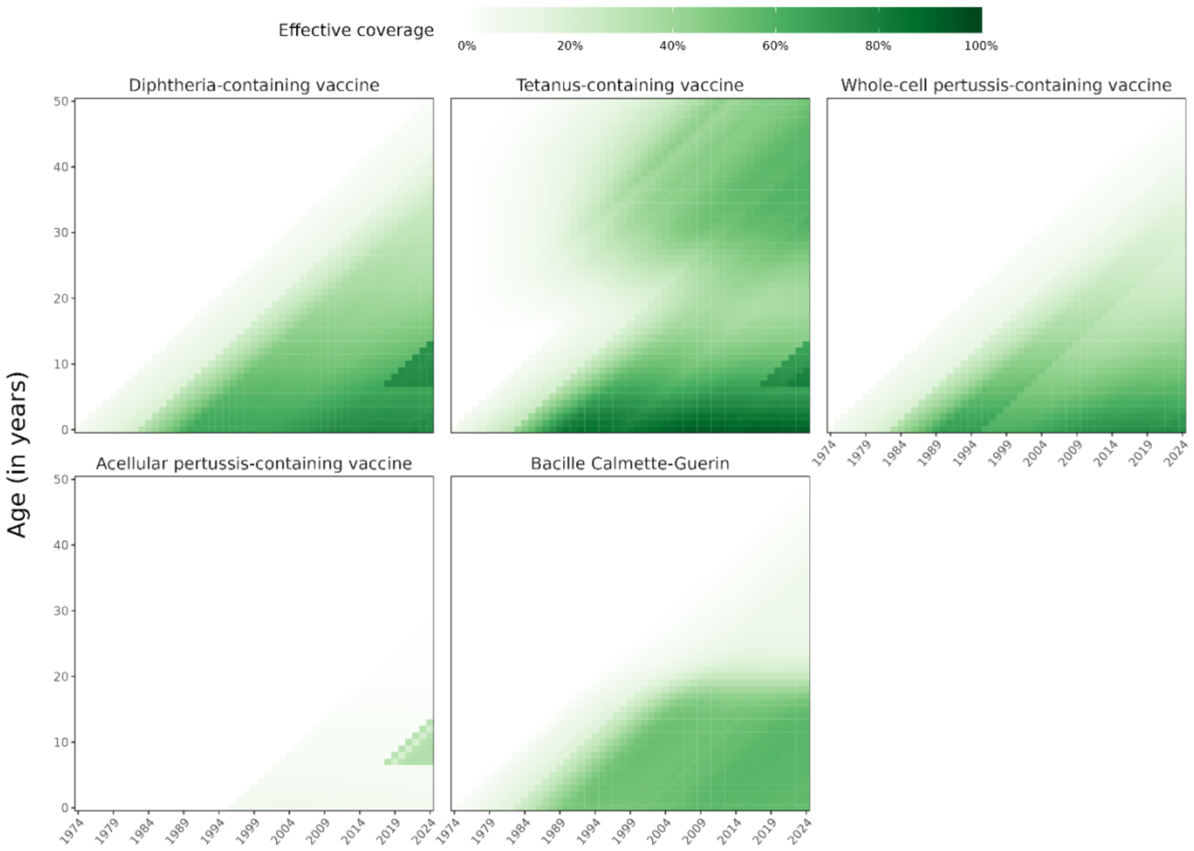

Figure S10 *Effective vaccine coverage for each vaccine.*

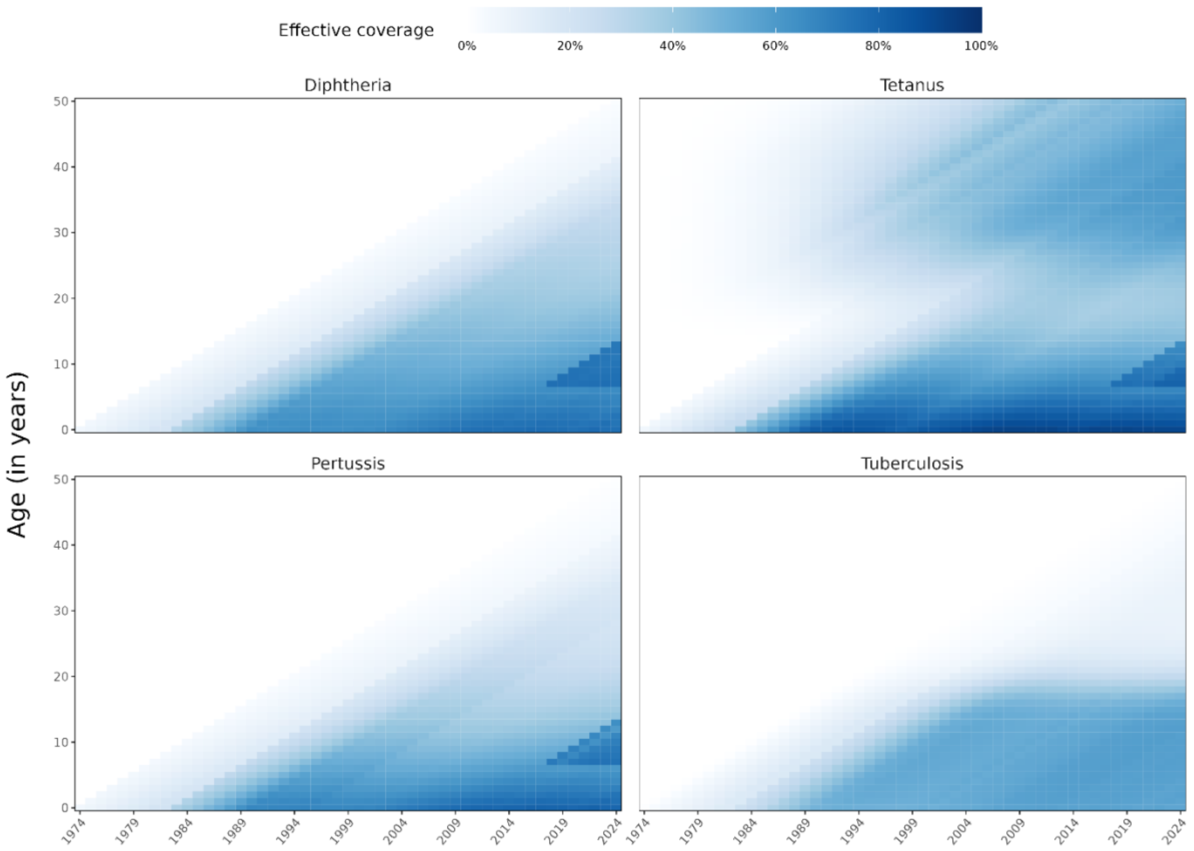

Figure S11 *Effective vaccine coverage aggregated for each disease.*

It remains to calculate disease burden averted using the disease-specific effective vaccine coverage values illustrated in Figure S10 in conjunction with disease-attributable mortality or mortality as estimated by GBD.[12] Disease-attributable mortality and morbidity estimates, as reported by GBD, are presented at the aggregated global level with broad age stratifications in Figure S12. Here we derive the model for estimating deaths averted. The model for DALYs averted / LFH gained is equivalent.

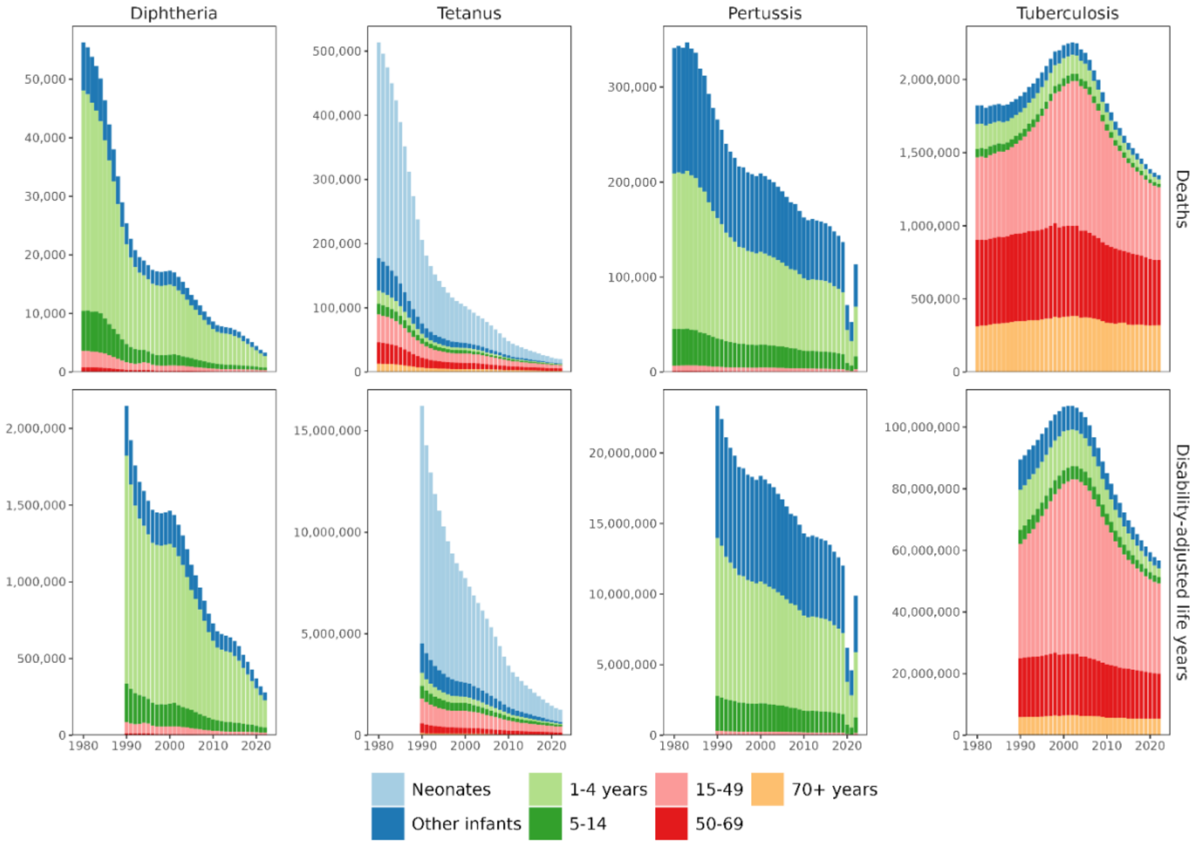

Figure S12 *Estimates of the global burden of diphtheria, tetanus, pertussis and TB in terms of deaths and disability-adjusted life years, by age and over time (Source: Global Burden of Disease study).*

Let $d_k(y, a)$ be the number of disease-specific deaths in Member State k, year y, and age a as reported by GBD. We then estimate the equivalent number of disease-specific deaths in the absence of vaccination $w_{d,k}$ using:

$$w_{d,k}(y, a) = \frac{d_k(y, a)}{1 - e_{d,k}(y, a)}$$

We then calculate the number of deaths averted $a_{d,k}$ as:

$$\begin{aligned} a_{d,k}(y, a) &= w_{d,k}(y, a) - d_k(y, a) \\ &= d_k(y, a)\left(\frac{1}{1 - e_{d,k}(y, a)} - 1\right) \end{aligned}$$

The total number of vaccine-attributable deaths averted for disease d in Member State k, denoted $A_{d,k}$, is then given by:

$$A_{d,k} = \sum_{y=1974}^{2024} \sum_{a=0}^{100} a_{d,k}(y, a)$$

Global, age-aggregated outcomes of deaths averted for each of the four diseases is presented in Figure S13 alongside GBD-derived estimates of disease-specific deaths. Note that the blue curves represent total values presented in Figure S12 The corresponding results for DALYs averted / YFH gained is presented in Figure S14. We here remark on several observations from these results. We find a substantial effect of vaccination on tetanus mortality. The historical age profile of tetanus – as estimated by GBD – reports a substantial burden of mortality and morbidity in neonatal infants (those under 4 weeks of age) (Figure S12). This age-structure of disease burden coupled with increasing vaccine coverage among pregnant women and a moderate protective effect of maternal immunity on neonates (Figures S9 and S11) results in an expected large impact. Conversely, the effect of BCG on tuberculous mortality is relatively modest. The age profile of tuberculous mortality burden over the past 50 years is much more heavily concentrated in older adults, with over half of deaths in those above 50 years of age (Figure S12). This age-structure, coupled with a vaccine that has a modest initial vaccine efficacy that has decayed to low levels after approximately 20 years (Figure S9) leads to a relatively modest expected impact of vaccination on disease-specific deaths. These two prominent outcomes lead to two conclusions relevant for the future: 1) maternal vaccination against tetanus has great potential to continue saving lives, and 2) innovations in tuberculosis vaccine development for vaccines efficacious in adolescents and adults have considerable scope to prevent future public heath burden. We also find a relatively low absolute effect of vaccine impact on averting diphtheria-related deaths. This finding is driven by a low estimated burden of disease in the vaccination era (Figure S12), and can be considered a conservative estimate that is likely an artefact of the relatively simplistic static modelling approach taken.

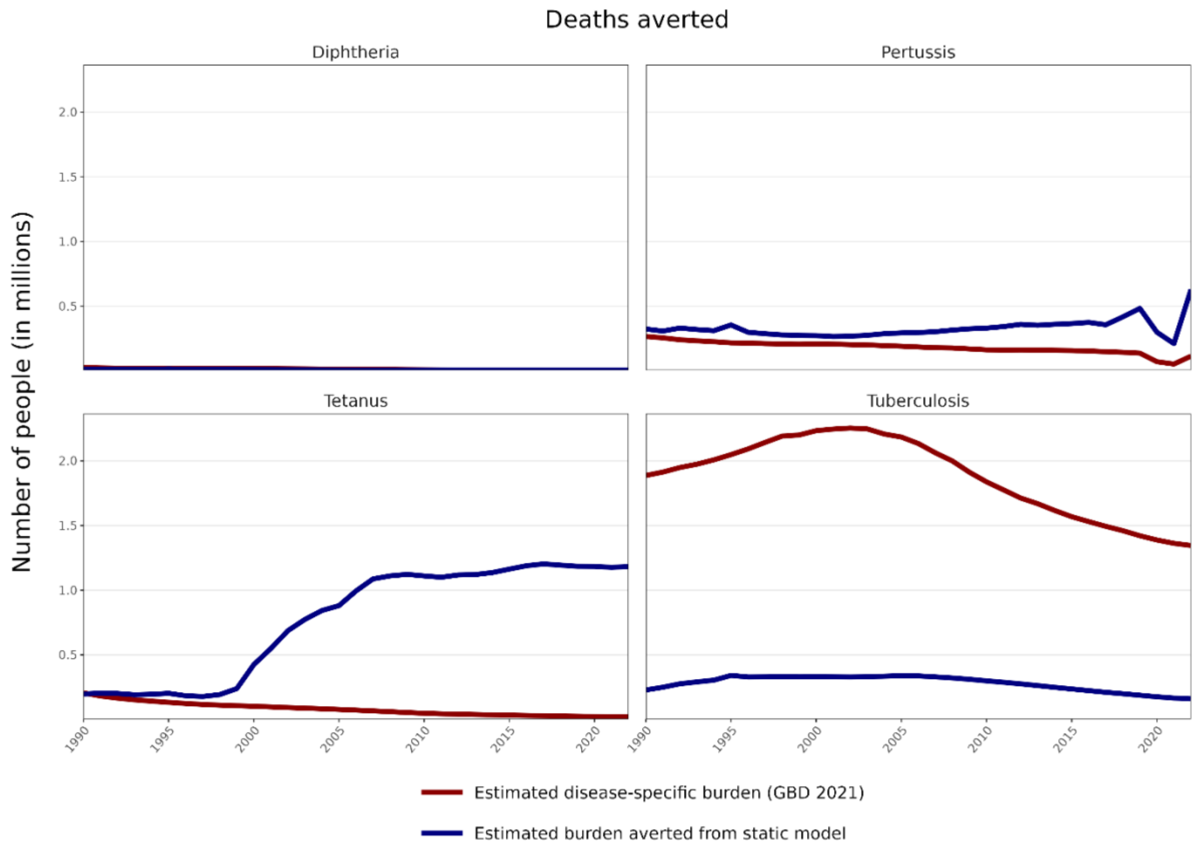

Figure S13 *Estimated disease-specific deaths and deaths averted through vaccination.*

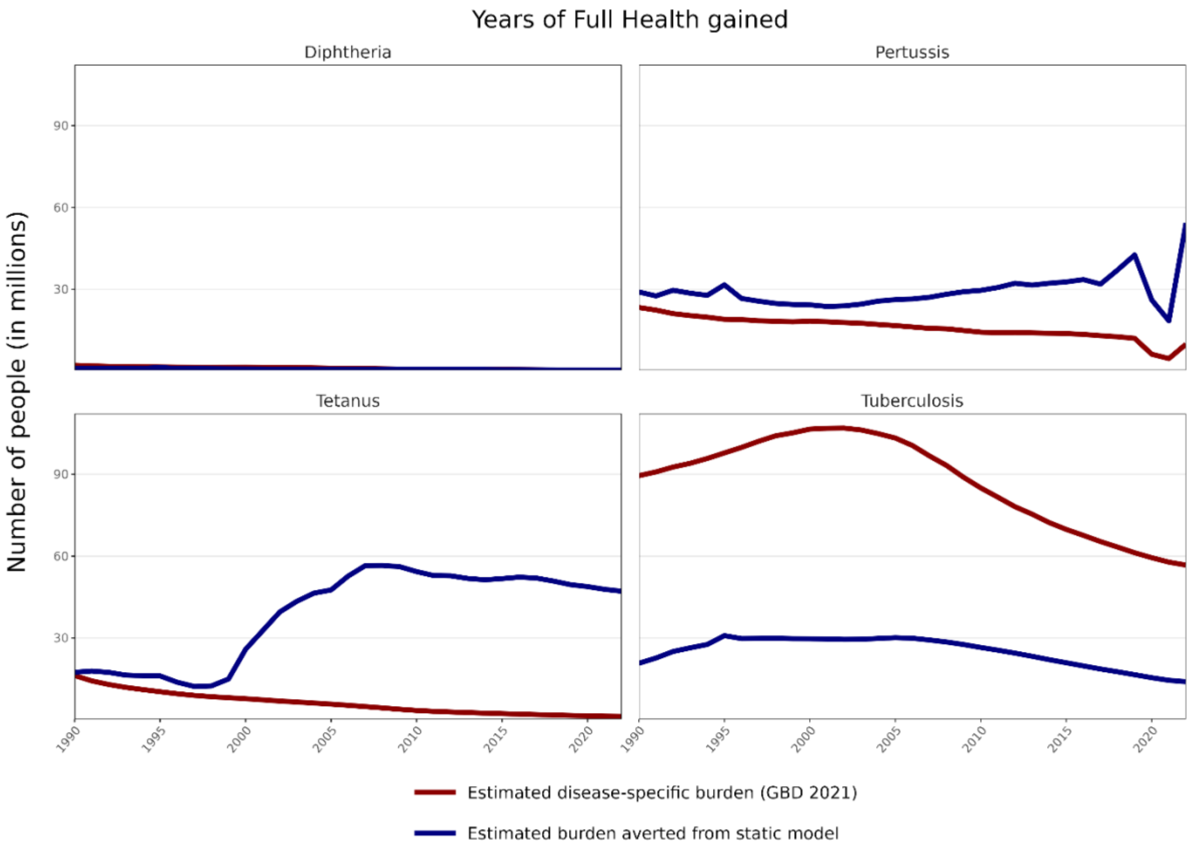

Figure S14 *Estimated disease-specific years of full health gained through vaccination.*

Extension of model estimates

Geographical imputation

To impute vaccine impact in Member States outside the scope of the VIMC, we fitted time series regression models with the outcome of deaths averted and YFH gained for each disease-vaccine-activity (vaccine dose number in routine programmes or supplementary immunization activities) in each Member State where VIMC estimates were available. Disease-vaccine-activities with fewer than 10 Member States were omitted as the sample was insufficient for extrapolation to other settings.

An initial range of predictor variables was selected, encompassing known covariates of vaccination impact, reported in a consistent way globally, over time. Time series regression models evaluate the relationship between the time series of the predictor and the time series of the outcome variable. For predictors where we had prior knowledge of a time-lagged effect, we included offset terms, as summarised in Table S8.

Table S8 *Predictor variables included in the model selection for geographical imputation.*

Variable	Description	Source
Vaccination coverage (current year, y)	Proportion of the population fully vaccinated, according to the study definition of a fully vaccinated person, in the same calendar year as the outcome variable	As per study
Vaccination coverage (y-1)	As vaccination coverage, with lag of 1 year	As per study
Vaccination coverage (y-2)	As vaccination coverage, with lag of 2 years	As per study
Vaccination coverage (y-3)	As vaccination coverage, with lag of 3 years	As per study
Vaccination coverage (y-4)	As vaccination coverage, with lag of 4 years	As per study
Stunting	Proportion of under-fives falling below minus 2 standard deviations (moderate and severe) and minus 3 standard deviations (severe) from the median height-for-age of the reference population	UNICEF[79]
Maternal mortality	Maternal deaths from pregnancy or childbirth per 100,000 live births	UNICEF[80]
Basic water	Proportion of population with access to at least a basic water source	Gapminder Foundation[81]
Basic sanitation	Proportion of population with access to at least basic sanitation	Gapminder Foundation[81]
Male adult literacy	Proportion of males aged 15 years and above who can read and write	World Bank[82]
Female adult literacy	Proportion of females aged 15 years and above who can read and write	World Bank[83]

Attended births	Proportion of births attended by skilled health workers	Gapminder Foundation[81]
Gini coefficient (current year, y)	Statistical dispersion of income	Gapminder Foundation[81]
Gini (y-1)	As Gini, with lag of 1 year	Gapminder Foundation[81]
Gini (y-2)	As Gini, with lag of 2 years	Gapminder Foundation[81]
Gross domestic product (current year, y)	GDP *per capita* in constant purchasing power parity US dollars	Gapminder Foundation[81]
GDP (y-1)	As GDP, with lag of 1 year	Gapminder Foundation[81]
GDP (y-2)	As GDP, with lag of 2 years	Gapminder Foundation[81]
Private health spending (current year, y)	Private share of total health spending	Gapminder Foundation[81]
Private health spending (y-1)	As private health spending, with lag of 1 year	Gapminder Foundation[81]
Private health spending (y-2)	As private health spending, with lag of 2 years	Gapminder Foundation[81]
Health spending (current year, y)	Total health spending *per capita*	Gapminder Foundation[81]
Health spending (y-1)	As health spending, with lag of 1 year	Gapminder Foundation[81]
Health spending (y-2)	As health spending, with lag of 2 years	Gapminder Foundation[81]

Where predictor data were temporally incomplete, we imputed missing values using linear models with a trend component (*forecast* package for R; Hyndman and Khandakar). Since the number of potential predictor combinations was large ($24! = 6 * 10^{23}$) we used a phased approach for model selection. In each phase, we used a corrected Akaike Information Criterion (AICc) to compare models, both

within the group and with those from the previous phase. The exercise was conducted for each disease-vaccine-activity combination in each Member State for which VIMC estimates of vaccine impact were available:

1. In the first phase, the AICc was used to assess how many lagged years of vaccination coverage should be included.

2. Secondly, we assessed the 11 single non-coverage predictors (stunting, maternal mortality, basic water, basic sanitation, male adult literacy, female adult literacy, attended births, Gini coefficient, GDP, total health spending, private health spending) and 55 pairwise combinations of group 2 predictors in combination with the best choice of vaccination coverage inclusion from phase 1.

3. Next, we assessed whether the 66 models in phase 2 could be improved by removing one or more years of lagged vaccination coverage

4. Where predictor combinations included the Gini coefficient, GDP, total health spending or private health spending, we assessed whether a model including lagged values of these variables would be preferable.

5. Finally, we assessed whether the 55 models that already included two non-coverage predictors could be improved by adding a third and, if so, a fourth. In practise, it was unnecessary to continue beyond this point.

The outcome of this stepwise model selection approach was a chosen model for each disease-vaccine-activity in each VIMC Member State. To avoid complexity (and noting that this may in itself be a form of over-fitting), we grouped Member States and selected the most common model choice for the group. Our baseline approach was to group Member States by WHO region but we conducted a sensitivity analysis grouping by current World Bank income level and by bands of current DTP vaccination coverage.

The model fit to observed vaccination impact from this approach is shown in Figures S15 and S16.

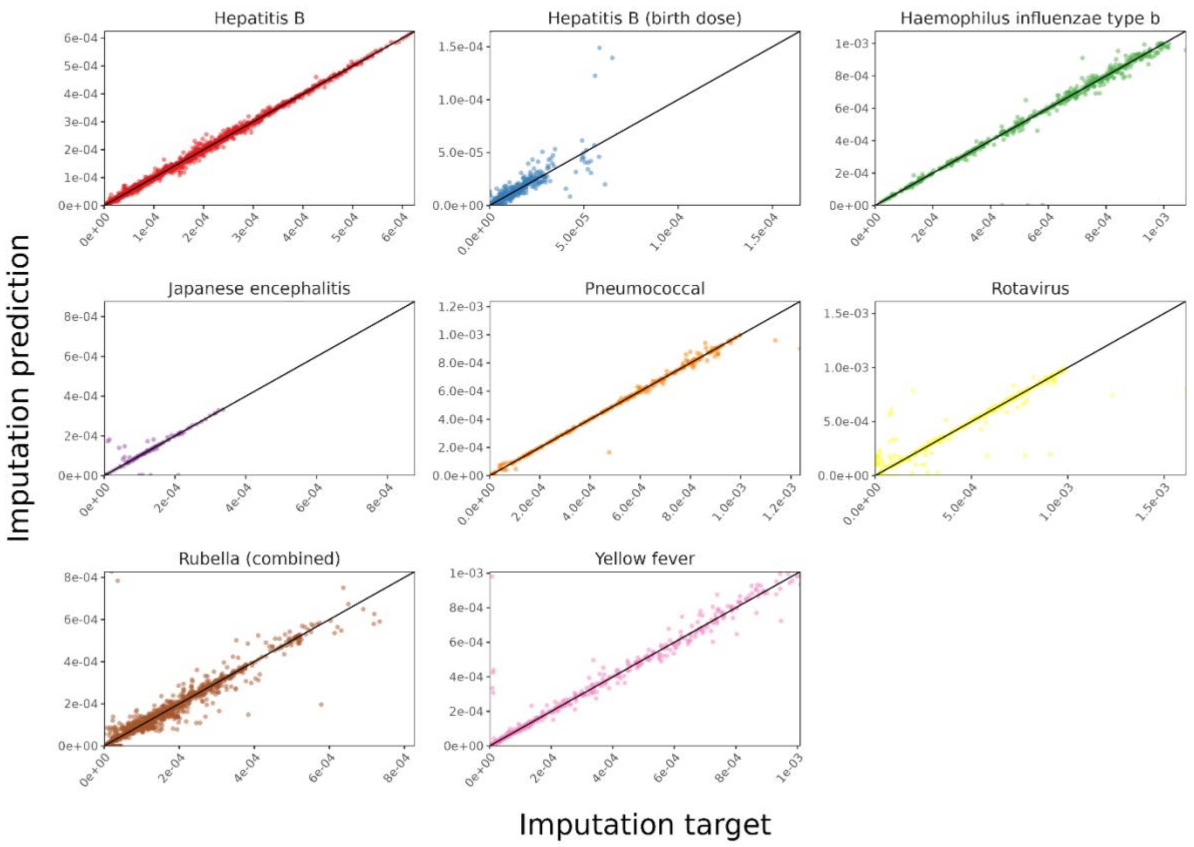

Figure S15 *Fit of time series regression models to observed vaccination impact for each disease-vaccine-activity with sufficient observations.*

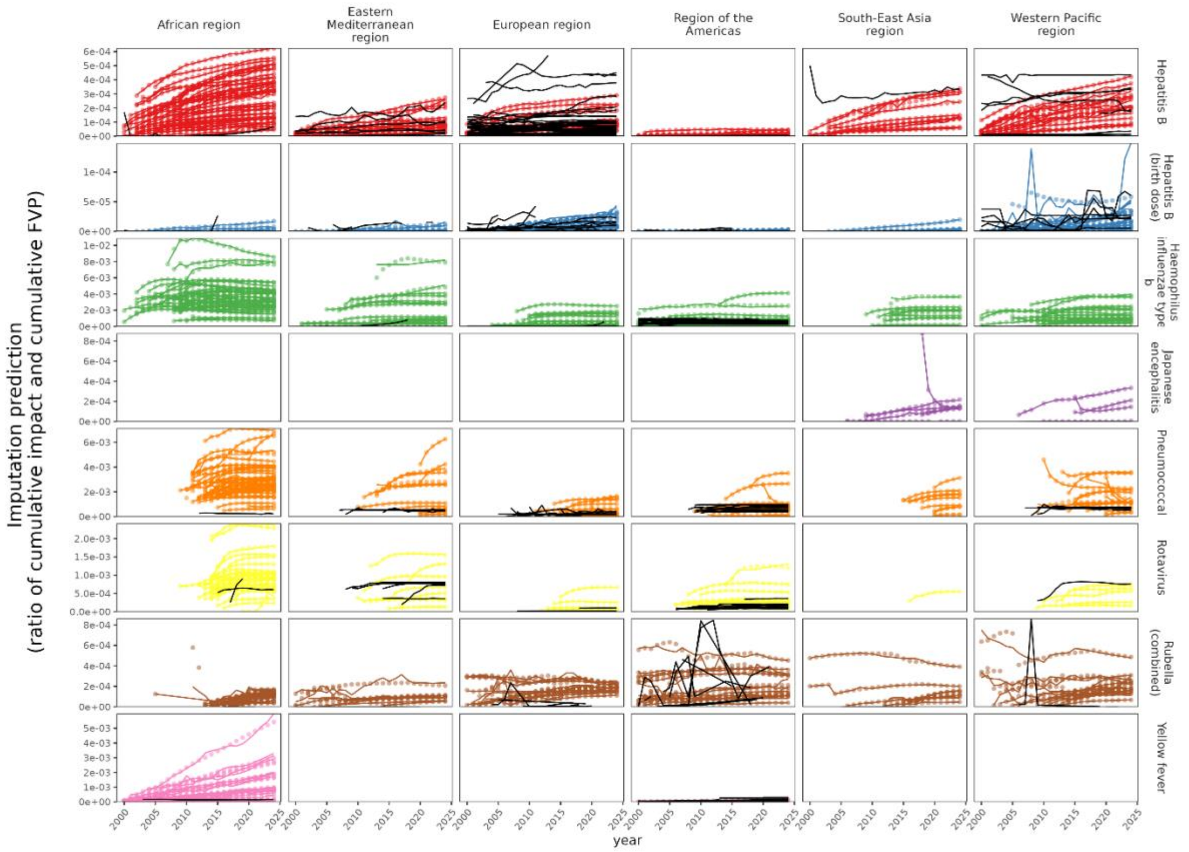

Figure S16 *Fit of time series regression models, plotted by disease-vaccine-activity and WHO region. Points represent 'data'; that is, transmission model estimated cumulative deaths averted divided by cumulative FVP. The corresponding coloured lines represent the regression model fit, grouped by Member State. Black lines represent the imputed ratio of cumulative deaths averted and cumulative FVP for Member States without transmission model estimates.*

For Member States without primary estimates of vaccination impact we used the selected model for their grouping again using WHO region in the baseline case and conducting a sensitivity analysis of World Bank income level and band of DTP vaccination coverage, using the group median of fitted predictor coefficients to inform the model.

Being conscious that the geographical imputation approach is, in effect, an extrapolation from VIMC to non-VIMC Member States, known to be contextually different, we assessed the validity of our estimates against observed measures of vaccination impact in the imputed Member States, where available.

Non-linear impact functions and temporal extrapolation

Vaccine impact derived using form 2 (VIMC modelling portfolio, eight pathogens) and form 3 (static models, four pathogens) represent only a subset of the 50 year timeframe of this analysis; 2000-2024 for form 2 and 1980-2021 for form 3. For several of the relevant pathogens, there exists vaccine coverage outside of the directly modelled temporal scope, for which we must extrapolate expected vaccine impact.

Over a short period, it may be reasonable to assume that the per-FVP impact of vaccination may hold constant over time. That is, for a given vaccine each FVP averts a consistent number of deaths (or gains a consistent number of YFH) year on year. However, over a long-term period – such as the 50 years considered in this analysis – such an assumption may not necessarily be suitable. We here offer some justification for this reasoning. Depending on the dynamics of the pathogen and properties of the vaccine itself, two complimentary effects of vaccination may be observed. First, an individual-level benefit that may prevent disease and/or death. Second, a population-level benefit that may break transmission chains (due to reduced bacterial/viral load in infected people and/or due to infection/transmission blocking effects of the vaccine). As vaccine coverage increases, maximal population-level benefits may become realised. Thus, over a given coverage threshold – commonly termed the 'herd immunity threshold' – the proportional gain of population benefit relative to individual benefit decreases. As such, the impact per FVP may begin to saturate for high coverage levels. We stress, however, that decreasing coverage would similarly be expected to result in a non-linear impact per drop in FVP; larger negative effects would occur as coverage falls back below herd immunity threshold. Moreover, there may also exist a growth in impact per FVP, especially in the early stages in vaccine rollout. As more of the population are vaccinated (up to a certain threshold), a pathogen may circulate less and therefore a proportional increase in population level benefit over individual level benefit may be observed.

With this reasoning considered, to accurately back or forward project vaccine impact per FVP beyond the directly modelled scope, we hypothesize that cumulative per-FVP impact may follow one of four functional forms: linear growth, exponential growth, logarithmic growth (saturating at high levels of coverage), or sigmoidal growth (initial exponential growth followed smoothly by saturating logarithmic growth). Table S9 describes the four functional forms used in more detail. All functions pass through the zero FVP – zero impact origin by construction.

Table S9 *Functions evaluated for impact factor model selection.*

Function name	Equation	Number of parameters	Description
Linear gradient	$y = p_1 x$	1	Straight line through the origin. Gradient represents an 'impact factor'.
Logarithmic growth	$y = \frac{p_1}{1 + e^{-p_2 x}} - {p_1}/{2}$	2	Logarithmic growth function that saturates.
Exponential growth	$y = p_1 e^{p_2 x} - p_1$	2	Exponential growth that continues to rise.

Sigmoidal growth	$y = p_1\left(1 - \frac{1}{1 + \left(x/p_2\right)^{p_3}}\right)$	3	Initial exponential rise smoothly continuing into logarithmic saturation. An ‘S’ shape.

Where the number of modelled cumulative FVP – cumulative deaths averted data is sufficient, each functional form is fitted for each disease-vaccine-activity and Member State combination. Formally, let k be the number of cumulative FVP – cumulative death averted data points for a given disease-vaccine-activity for a given Member State. An artificial data point is appended at the origin (that is, zero FVP implies zero vaccine impact), such that each function is fit to $k + 1$ coordinates. For a function with n parameters, we attempt to fit said function to the data only if $n \leq k$. Thus, in the extreme case of $k = 1$, only the linear gradient function is fitted.

For each disease-vaccine-activity and Member State combination, a Monte Carlo Markov Chain (MCMC) algorithm was used to derive posteriors for all fitted function parameters. The priors used for the MCMC process were derived by an Adaptive Stochastic Descent optimsation algorithm, with the result of this optimisation informing the mean of each parameter prior. The prior for each parameter was assumed to be Gaussian distributed with a unit standard deviation. The MCMC algorithm was run ten times for each disease-vaccine-activity-Member State instance to ensure the resulting chains converged to the same globally optimal result. Posterior samples were then generated by randomly selecting a subset from the combination of the ten MCMC chains. Finally, a corrected Akaike Information Criterion (AICc) score was used to select the most appropriate functional form for each disease-vaccine-activity and Member State combination. The AICc model selection criteria helps to ensure the best fitting functional form is selected, whilst reducing the probability of selecting a function that over-fits the data. Figure S17 illustrates the data (presented as points) and resulting fitted functional form (lines) selected for each disease-vaccine-activity. Figure S18 shows the proportion of Member States for which each functional form was selected, for each disease-vaccine-activity.

Using these fitted relationships between vaccination coverage (in terms of cumulative FVP) and vaccine impact (in terms of cumulative deaths averted / cumulative YFH gained), we inferred vaccine impact either back or forward in time according to observed coverage in all cases where vaccine impact was not directly modelled.

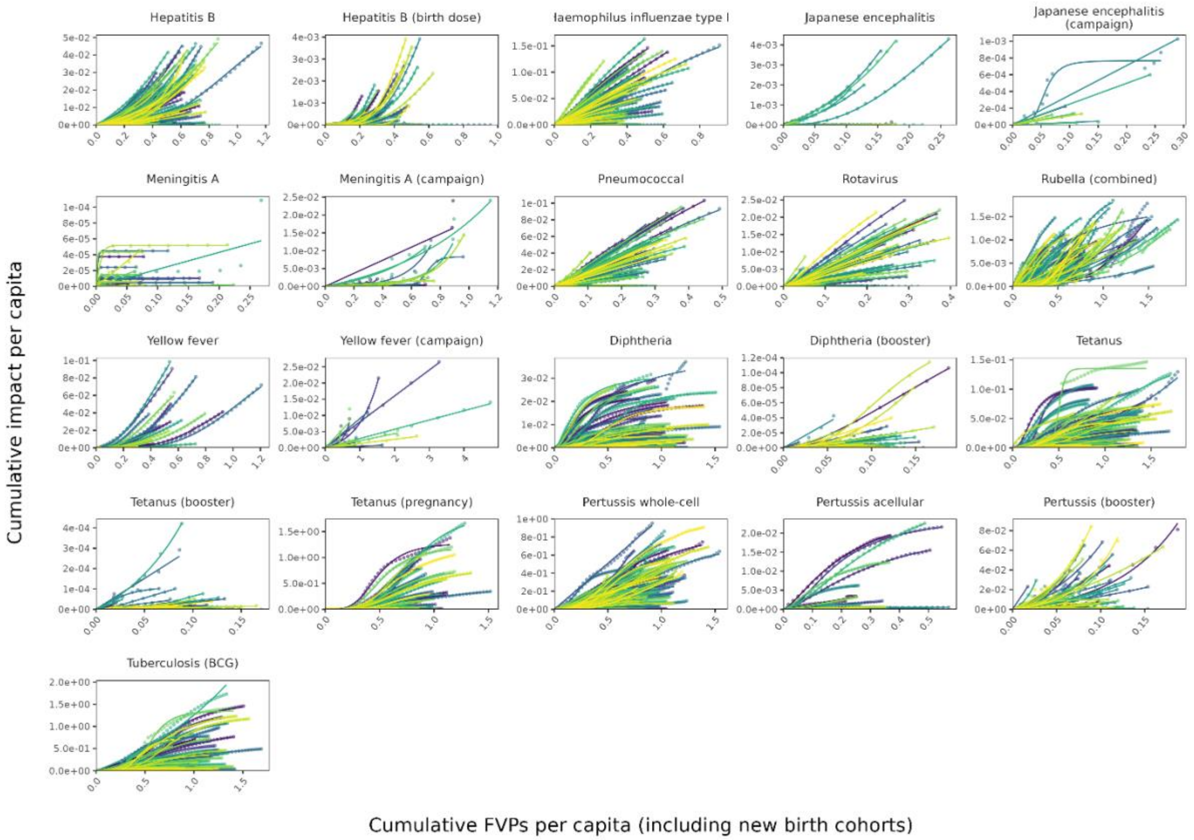

Figure S17 *Fitted functional representation of cumulative FVP against cumulative deaths averted per capita. Points represent 'data'; that is, outcomes from 1) transmission models, or 2) geographical imputation time series models. Lines represent impact function fits. Points and lines and grouped and coloured by Member State.*

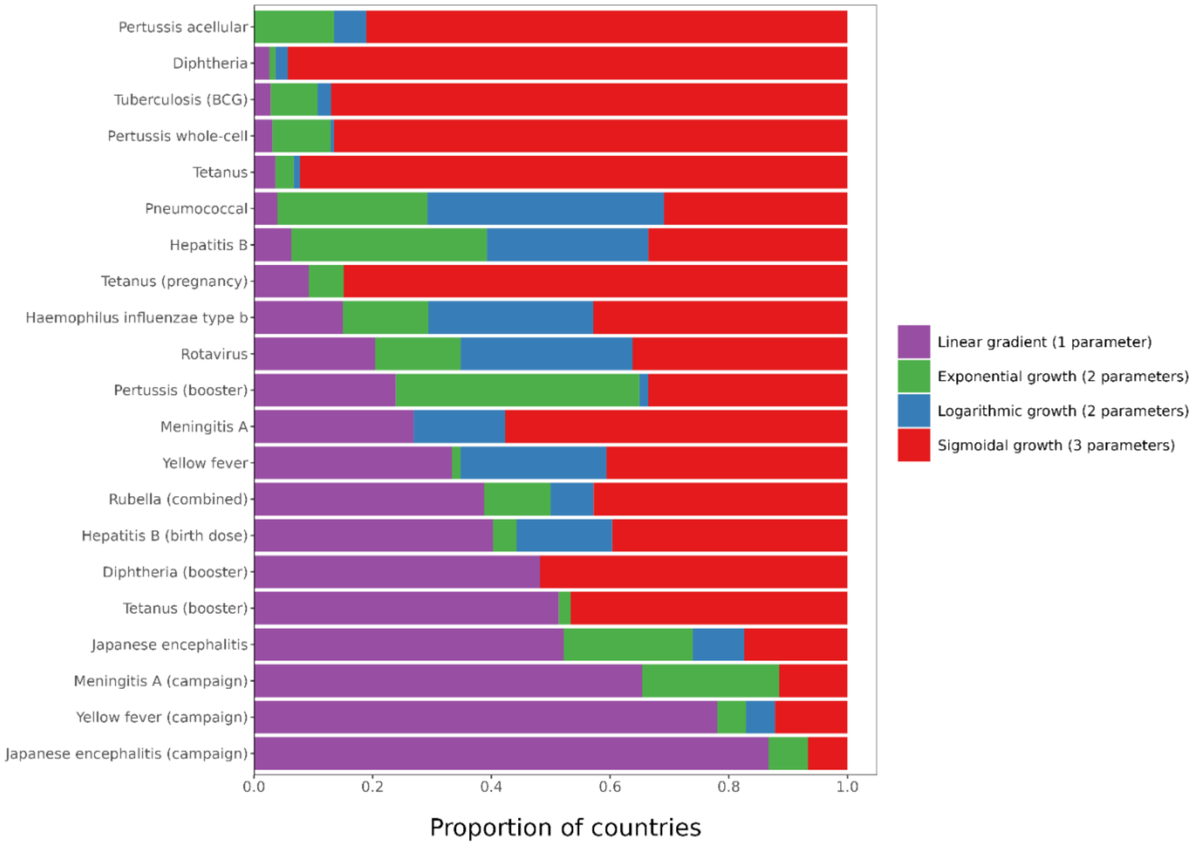

Figure S18 *Proportion of Member States for which each functional form was selected by disease-vaccine-activity.*

Uncertainty of estimates

It is important to appreciate that our uncertainty bounds relate only to the work of our modelling. They are not bounds around the veracity of the estimates themselves. It is not possible to propagate uncertainty at all levels of estimation, of all the hierarchical underlying models or of the values input into the models. For example, we took existing estimates of coverage and of population denominators as such, that is we took them to be true. We used these inputs to conduct our modelling, and we fit functional forms and derived Markov Chain Monte Carlo posteriors. In the process of deriving these posteriors we predefined the magnitude of the allowable uncertainty. In this sense the bounds are arbitrary. They broadly show the scale of uncertainty, but they should not be interpreted as a claim to where the edges of valid estimates possible lie. Bounds we produce do not describe the probability distribution of the true estimator under our assumptions, as bounds of regression say are usually interpreted. Put simply, the bounds are not as big as they should be given the multiple levels of uncertainty, and do not carry the usual meaning.

The coverage estimates that derive from WIISE are those that are produced through the WHO and UNICEF Estimates of National Immunization Coverage (WUENIC). WUENIC numbers are not statistical estimands, and do not have uncertainty bounds. They are based on a logic model that applies predetermined rules to triangulate Member States' official reported coverage against

other sources of evidence, to achieve a final determination of national coverage. The methods for WUENIC are published, and also made available on the WUENIC site. WUENIC numbers form the input for many arising statistical or modelling efforts by academic institutions globally. More importantly they form the basis for country decision making and planning. Further methods, and relevant publications, are available here:

www.who.int/docs/default-source/immunization/immunization-coverage/wuenic_notes.pdf?sfvrsn=88ff590d_6

WPP estimates are produced by the UN Population Division. UNPD produce uncertainty bounds for prospective population projections, but not for past population figures, the latter being relevant for our present analysis. To be sure even retrospective numbers are derived from hierarchical Bayesian models that do incorporate uncertainty bounds, for example with respect to inter alia fertility rate assumptions or sex ratio at birth, cause of death data, international migration, et cet. See for example Liu, P., and A.E. Raftery (2020). Accounting for uncertainty about past values in probabilistic projections of the total fertility rate for most Member States. The annals of applied statistics, vol. 14, No. 2, pp. 685-705. WPP methods are described here:

https://population.un.org/wpp/Publications/Files/WPP2022_Methodology.pdf

Interested readers who seek to extend on our work, (we have made public our data and code) will of course need to grapple with the same issues, as we have done.

Accounting for potential double counting

Since we estimate the deaths averted separately by a number of disease-vaccine-activities, we assessed the potential impact of double counting. That is, a life saved from measles may later be saved from invasive pneumococcal disease but should not count as two lives and falsely inflate the overall rate of reduced mortality.

We used a Bernoulli process to provide an estimate of the potential scale of this effect. Although the Bernoulli method for competing risks provides a lower bound, it is accepted as the most robust approach for finding an approximate value. The equation is:

$$mortality_{combined} = 1 - \prod_{dva}(1 - mortality_{dva})$$

By calculating the combined mortality in the presence and absence of vaccination, we quantified the potential impact of double counting on the number of deaths averted. For infants aged 12 months and younger, 355,000 deaths may have been counted twice, 0.343% of the total. For the overall population, the number is lower since the rate of deaths averted by each disease-vaccine-activity is lower. Making the limiting assumption that risk in the population is homogenous, it is much less likely that the same person will be affected by two diseases. The potential number of deaths counted twice in the overall population is 25,000 which equates to 0.01% of the total.

Code library and reproducibility

The open source code library for this analysis is publicly available from the World Health Organization GitHub repository:

https://github.com/WorldHealthOrganization/epi50-vaccine-impact

For longevity of the code used to generate all results and figures presented here, all files can be downloaded from Zenodo (DOI: 10.5281/zenodo.10980462):

https://zenodo.org/doi/10.5281/zenodo.10974443

All input data and configuration files required to reproduce this analysis in full are also open source and contained within this repository. Code is written in the R programming language. An internet connection is required to run the analysis, which can be run on both Windows and UNIX operating systems.

Supplementary References

1. World Health Assembly. WHO expanded programme on immunization. 1974. https://iris.who.int/handle/10665/92778 (accessed 12 March 2024).
2. Chan M. The Contribution of Immunization: Saving Millions of Lives, and More. *Public Health Reports* 2014; **129**(5_suppl3): 7-8.
3. World Health Organization. WHO/UNICEF estimates of national immunization coverage. 2023. https://www.who.int/teams/immunization-vaccines-and-biologicals/immunization-analysis-and-insights/global-monitoring/immunization-coverage/who-unicef-estimates-of-national-immunization-coverage (accessed 12 March 2024).
4. World Health Organization. WHO Immunization Data Portal 2023. https://immunizationdata.who.int/.
5. World Health Organization. WHO IVB Supplementary Immunisation Activity database. 2023.
6. Toor J, Echeverria-Londono S, Li X, et al. Lives saved with vaccination for 10 pathogens across 112 countries in a pre-COVID-19 world. *Elife* 2021; **10**.
7. United Nations Department of Economic and Social Affiars Population Division. The 2022 Revision of World Population Prospects. 2022. https://population.un.org/wpp/.
8. Eilertson KE, Fricks J, Ferrari MJ. Estimation and prediction for a mechanistic model of measles transmission using particle filtering and maximum likelihood estimation. *Statistics in Medicine* 2019; **38**(21): 4146-58.
9. Verguet S, Johri M, Morris SK, Gauvreau CL, Jha P, Jit M. Controlling measles using supplemental immunization activities: A mathematical model to inform optimal policy. *Vaccine* 2015; **33**(10): 1291-6.
10. Badizadegan K, Kalkowska DA, Thompson KM. Polio by the Numbers—A Global Perspective *The Journal of Infectious Diseases* 2022; **226**(8): 1309-18.
11. Carter A, Msemburi W, Sim SY, et al. Modeling the impact of vaccination for the immunization Agenda 2030: Deaths averted due to vaccination against 14 pathogens in 194 countries from 2021 to 2030. *Vaccine* 2023.
12. Institute for Health Metrics and Evaluation (IHME). Global Burden of Disease (GBD). https://www.healthdata.org/research-analysis/gbd (accessed 16 April 2024).
13. World Health Organization. Immunization and vaccines related implementation research advisory committee (IVIR-AC). https://www.who.int/groups/immunization-and-vaccines-related-implementation-research-advisory-committee (accessed 17 March 2024).
14. Immunization Agenda 2030. IA2030 Technical Progress Report to the Strategic Advisory Group of Experts (SAGE) September 2023, 2023.
15. World Health Organization. Progress and challenges with Achieving Universal Immunization Coverage. 2023 (accessed 12 March 2024).
16. World Health Organization. BCG vaccines: WHO position paper – February 2018, 2018.
17. Clark RA, Mukandavire C, Portnoy A, et al. The impact of alternative delivery strategies for novel tuberculosis vaccines in low-income and middle-income countries: a modelling study. *The Lancet Global Health* 2023; **11**(4): e546-e55.
18. Jit M, Hutubessy R, Png ME, et al. The broader economic impact of vaccination: reviewing and appraising the strength of evidence. *BMC Medicine* 2015; **13**(1): 209.
19. Echeverria-Londono S, Li X, Toor J, et al. How can the public health impact of vaccination be estimated? *BMC Public Health* 2021; **21**(1): 2049.
20. Brisson M, Kim JJ, Canfell K, et al. Impact of HPV vaccination and cervical screening on cervical cancer elimination: a comparative modelling analysis in 78 low-income and lower-middle-income countries. *The Lancet* 2020; **395**(10224): 575-90.

21. World Health Organization. Cervical Cancer Elimination Initiative. https://www.who.int/initiatives/cervical-cancer-elimination-initiative#cms.
22. Cornejo S, Chevez A, Ozturk M, et al. [The Pan American Health Organization's Revolving Fund for access to vaccines: 43 years responding to the regional immunization programO Fundo Rotativo para Acesso a Vacinas da Organização Pan-Americana da Saúde: 43 anos respondendo ao Programa Regional de Imunizações]. *Rev Panam Salud Publica* 2023; **47**: e50.
23. UNICEF. Moving with the times: 1980–1988: Discover the importance of data and research in UNICEF’s efforts for child survival and development. https://www.unicef.org/stories/learning-experience-19801988 (accessed 16 March 2024).
24. O'Brien K. From the expanded to the essential programme on immunization: Achievements of the last 50 years and inspirations for the next 50. IVB Director’s Report to SAGE., 2024.
25. Institute of Medicine. The Children's Vaccine Initiative: Achieving the Vision. Washington, DC: The National Academies Press; 1993.
26. World Health Organization. Strategic Advisory Group of Experts (SAGE) on Immunization https://www.who.int/groups/strategic-advisory-group-of-experts-on-immunization/about (accessed 16 March 2024).
27. Gavi the Vaccine Alliance. A History of Gavi, the Vaccine Alliance. 2023. p. https://www.youtube.com/watch?v=7QTf5CPC1Tw.
28. Gavi the Vaccine Alliance. ADIPs and Hib Initiative evaluation. 2019. https://www.gavi.org/our-impact/evaluation-studies/adips-and-hib-initiative-evaluation (accessed 16 March 2024).
29. Gavi the Vaccine Alliance. Pneumococcal AMC. 2021. https://www.gavi.org/investing-gavi/innovative-financing/pneumococcal-amc (accessed 16 March 2024).
30. PATH. The Meningitis Vaccine Project: A groundbreaking partnership. 2015. https://www.path.org/our-impact/articles/about-meningitis-vaccine-project/ (accessed 17 March 2024).
31. World Health Organization. Malaria vaccine implementation programme. https://www.who.int/initiatives/malaria-vaccine-implementation-programme (accessed 16 March 2024).
32. Coalition for Epidemic Preparedness Innovations. CEPI. https://cepi.net/ (accessed 17 March 2024).
33. Immunization Agenda 2030. Immunization Agenda 2030. 2024. https://www.immunizationagenda2030.org/.
34. World Health Organization. The Access to COVID-19 Tools (ACT) Accelerator. 2024. https://www.who.int/initiatives/act-accelerator.
35. World Health Organization. The Big Catch-Up: An Essential Immunization Recovery Plan for 2023 and Beyond: World Health Organization (WHO), United Nations Children’s Fund (UNICEF), 2023.
36. The Vaccine Impact Modelling Consortium (VIMC). Data visualisations. 2019
37. Auzenbergs M, Fu H, Abbas K, Procter SR, Cutts FT, Jit M. Health effects of routine measles vaccination and supplementary immunisation activities in 14 high-burden countries: a Dynamic Measles Immunization Calculation Engine (DynaMICE) modelling study. *The Lancet Global Health* 2023; **11**(8): e1194-e204.
38. The Polaris Observatory Collaborators. Global prevalence, treatment, and prevention of hepatitis B virus infection in 2016: a modelling study. *The Lancet Gastroenterology & Hepatology* 2018; **3**(6): 383-403.
39. Nayagam S, Thursz M, Sicuri E, et al. Requirements for global elimination of hepatitis B: a modelling study. *The Lancet Infectious Diseases* 2016; **16**(12): 1399-408.

40. de Villiers MJ, Gamkrelidze I, Hallett TB, Nayagam S, Razavi H, Razavi-Shearer D. Modelling hepatitis B virus infection and impact of timely birth dose vaccine: A comparison of two simulation models. *PLOS ONE* 2020; **15**(8): e0237525.
41. Goldstein ST, Zhou F, Hadler SC, Bell BP, Mast EE, Margolis HS. A mathematical model to estimate global hepatitis B disease burden and vaccination impact. *International Journal of Epidemiology* 2005; **34**(6): 1329-39.
42. Walker N, Tam Y, Friberg IK. Overview of the Lives Saved Tool (LiST). *BMC Public Health* 2013; **13**(3): S1.
43. Clark A, Black R, Tate J, et al. Estimating global, regional and national rotavirus deaths in children aged <5 years: Current approaches, new analyses and proposed improvements. *PLOS ONE* 2017; **12**(9): e0183392.
44. Quan TM, Thao TTN, Duy NM, Nhat TM, Clapham H. Estimates of the global burden of Japanese encephalitis and the impact of vaccination from 2000-2015. *eLife* 2020; **9**: e51027.
45. van den Hurk AF, Ritchie SA, Mackenzie JS. Ecology and Geographical Expansion of Japanese Encephalitis Virus. *Annual Review of Entomology* 2009; **54**(Volume 54, 2009): 17-35.
46. Longbottom J, Browne AJ, Pigott DM, et al. Mapping the spatial distribution of the Japanese encephalitis vector, Culex tritaeniorhynchus Giles, 1901 (Diptera: Culicidae) within areas of Japanese encephalitis risk. *Parasites & Vectors* 2017; **10**(1): 148.
47. Karachaliou A, Conlan AJK, Preziosi M-P, Trotter CL. Modeling Long-term Vaccination Strategies With MenAfriVac in the African Meningitis Belt. *Clinical Infectious Diseases* 2015; **61**(suppl_5): S594-S600.
48. Jackson ML, Diallo AO, Médah I, et al. Initial validation of a simulation model for estimating the impact of serogroup A Neisseria meningitidis vaccination in the African meningitis belt. *PLOS ONE* 2018; **13**(10): e0206117.
49. Pitzer VE, Atkins KE, de Blasio BF, et al. Direct and Indirect Effects of Rotavirus Vaccination: Comparing Predictions from Transmission Dynamic Models. *PLOS ONE* 2012; **7**(8): e42320.
50. Metcalf CJE, Lessler J, Klepac P, Cutts F, Grenfell BT. Impact of birth rate, seasonality and transmission rate on minimum levels of coverage needed for rubella vaccination. *Epidemiology and Infection* 2012; **140**(12): 2290-301.
51. Vynnycky E, Adams EJ, Cutts FT, et al. Using Seroprevalence and Immunisation Coverage Data to Estimate the Global Burden of Congenital Rubella Syndrome, 1996-2010: A Systematic Review. *PLOS ONE* 2016; **11**(3): e0149160.
52. Vynnycky E, Papadopoulos T, Angelis K. The impact of Measles-Rubella vaccination on the morbidity and mortality from Congenital Rubella Syndrome in 92 countries. *Human Vaccines & Immunotherapeutics* 2019; **15**(2): 309-16.
53. Garske T, Van Kerkhove MD, Yactayo S, et al. Yellow Fever in Africa: Estimating the Burden of Disease and Impact of Mass Vaccination from Outbreak and Serological Data. *PLOS Medicine* 2014; **11**(5): e1001638.
54. Gaythorpe KAM, Hamlet A, Jean K, et al. The global burden of yellow fever. *eLife* 2021; **10**: e64670.
55. Johansson MA, Vasconcelos PFC, Staples JE. The whole iceberg: estimating the incidence of yellow fever virus infection from the number of severe cases. *Transactions of The Royal Society of Tropical Medicine and Hygiene* 2014; **108**(8): 482-7.
56. Institute for Health Metrics and Evaluation (IHME). Diphtheria. https://www.healthdata.org/gbd/methods-appendices-2021/diphtheria (accessed 16 April 2024).
57. Institute for Health Metrics and Evaluation (IHME). Tetanus. https://www.healthdata.org/gbd/methods-appendices-2021/tetanus (accessed 16 April 2024).
58. Institute for Health Metrics and Evaluation (IHME). Pertussis (whooping cough). https://www.healthdata.org/gbd/methods-appendices-2021/pertussis-whooping-cough (accessed 16 April 2024).

59. Institute for Health Metrics and Evaluation (IHME). Tuberculosis. https://www.healthdata.org/gbd/methods-appendices-2021/tuberculosis (accessed 16 April 2024).
60. Kerr CC, Dura-Bernal S, Smolinski TG, Chadderdon GL, Wilson DP. Optimization by Adaptive Stochastic Descent. *PLOS ONE* 2018; **13**(3): e0192944.
61. Truelove SA, Keegan LT, Moss WJ, et al. Clinical and Epidemiological Aspects of Diphtheria: A Systematic Review and Pooled Analysis. *Clin Infect Dis* 2020; **71**(1): 89-97.
62. World Health Organization. Diphtheria vaccine: WHO position paper – August 2017, 2017.
63. Einarsdottir S, Sverrisdottir I, Vaht K, et al. Long-Term Immunity Against Tetanus and Diphtheria after Vaccination of Allogeneic Stem Cell Transplantation Recipients. *Transplantation and Cellular Therapy* 2023; **29**(4): 275.e1-.e5.
64. Hammarlund E, Thomas A, Poore EA, et al. Durability of Vaccine-Induced Immunity Against Tetanus and Diphtheria Toxins: A Cross-sectional Analysis. *Clin Infect Dis* 2016; **62**(9): 1111-8.
65. Bisgard KM, Rhodes P, Hardy IR, et al. Diphtheria toxoid vaccine effectiveness: A case-control study in Russia. *J Infect Dis* 2000; **181 Suppl 1**: S184-7.
66. Chen RT, Hardy IRB, Rhodes PH, Tyshchenko DK, Moiseeva AV, Marievsky VF. Ukraine, 1992: First assessment of diphtheria vaccine effectiveness during the recent resurgence of diphtheria in the former Soviet Union. *The Journal of Infectious Diseases* 2000; **181**(Supplement_1): S178-S83.
67. Alghounaim M, Alsaffar Z, Alfraij A, Bin-Hasan S, Hussain E. Whole-Cell and Acellular Pertussis Vaccine: Reflections on Efficacy. *Medical Principles and Practice* 2022; **31**(4): 313-21.
68. World Health Organization. Pertussis vaccines: WHO position paper – October 2010, 2010.
69. Luz PM, Struchiner CJ, Kim SY, et al. Modeling the cost-effectiveness of maternal acellular pertussis immunization (aP) in different socioeconomic settings: A dynamic transmission model of pertussis in three Brazilian states. *Vaccine* 2021; **39**(1): 125-36.
70. Schwartz KL, Kwong JC, Deeks SL, et al. Effectiveness of pertussis vaccination and duration of immunity. *Cmaj* 2016; **188**(16): E399-e406.
71. Domenech de Cellès M, Rohani P, King AA. Duration of Immunity and Effectiveness of Diphtheria-Tetanus-Acellular Pertussis Vaccines in Children. *JAMA Pediatr* 2019; **173**(6): 588-94.
72. World Health Organization. Tetanus vaccines: WHO position paper – May 2006, 2006.
73. Scobie HM, Khetsuriani N, Tohme RA, Acosta AM, Wassilak SGF, Orenstein WA. Chapter 59 - Tetanus Toxoid. In: Orenstein W, Offit P, Edwards KM, Plotkin S, eds. Plotkin's Vaccines (Eighth Edition). Philadelphia: Elsevier; 2023: 1117-41.e16.
74. Singh A, Pallikadavath S, Ogollah R, Stones W. Maternal tetanus toxoid vaccination and neonatal mortality in rural north India. *PLoS One* 2012; **7**(11): e48891.
75. Schofield FD, Tucker VM, Westbrook GR. Neonatal tetanus in New Guinea. Effect of active immunization in pregnancy. *Br Med J* 1961; **2**(5255): 785-9.
76. Blencowe H, Lawn J, Vandelaer J, Roper M, Cousens S. Tetanus toxoid immunization to reduce mortality from neonatal tetanus. *Int J Epidemiol* 2010; **39 Suppl 1**(Suppl 1): i102-9.
77. Harris RC, Dodd PJ, White RG. The potential impact of BCG vaccine supply shortages on global paediatric tuberculosis mortality. *BMC Medicine* 2016; **14**(1): 138.
78. Roy P, Vekemans J, Clark A, Sanderson C, Harris RC, White RG. Potential effect of age of BCG vaccination on global paediatric tuberculosis mortality: a modelling study. *The Lancet Global Health* 2019; **7**(12): e1655-e63.
79. UNICEF. Child Malnutrition. 2023. https://data.unicef.org/topic/nutrition/malnutrition/#status.
80. UNICEF. Maternal mortality. 2023.

81. The Gapminder Foundation. Open Numbers. Crowd-sourced harmonization of global & local statistics. https://github.com/open-numbers/ddf--gapminder--systema_globalis.
82. The World Bank. Literacy rate, adult male (% of males ages 15 and above).
83. The World Bank. Literacy rate, adult female (% of females ages 15 and above). https://data.worldbank.org/indicator/SE.ADT.LITR.FE.ZS.
